# Supplementary material for: Violence experience by perpetrator and associations with HIV/STI risk and infection: a cross-sectional study among female sex workers in Karnataka, south India
Source: BMJ Open. 2018 Sep 11;8(9):e021389. doi: 10.1136/bmjopen-2017-021389 (PMC6144389; doi:10.1136/bmjopen-2017-021389)
Supplement: Supplementary data [file bmjopen-2017-021389supp001.pdf]

Appendix A: IBBA violence questionnaire

Physical and sexual violence from a husband/main partner based on WHO operational definitions of violence	Q717b: A. Has any husband or main partner that you have lived with ever done following things to you?<br><br>a) pushed you, shaken you, or thrown something at you?<br><br>b) slapped or shoved you?<br><br>c) hit you with his fist or something else that could hurt you?<br><br>d) kicked you, dragged you or beat you up?<br><br>e) tried to choke you or burn you on purpose<br><br>f) threatened to use or actually used a knife, gun or any other weapon?<br><br>g) physically forced to have sex with him even when you did not want.<br><br>h) used threats of violence or rejection to forced you to have sex with him when you did not want to?<br><br>B. How often has this happened during the last 12 months: often, only sometimes, or not at all?
