# Supplementary material for: Violence experience by perpetrator and associations with HIV/STI risk and infection: a cross-sectional study among female sex workers in Karnataka, south India
Source: BMJ Open. 2018 Sep 11;8(9):e021389. doi: 10.1136/bmjopen-2017-021389 (PMC6144389; doi:10.1136/bmjopen-2017-021389)
Supplement: Author's manuscript [file bmjopen-2017-021389.draft_revisions.pdf]

BMJ Open is committed to open peer review. As part of this commitment we make the peer review history of every article we publish publicly available.

When an article is published we post the peer reviewers' comments and the authors' responses online. We also post the versions of the paper that were used during peer review. These are the versions that the peer review comments apply to.

The versions of the paper that follow are the versions that were submitted during the peer review process. They are not the versions of record or the final published versions. They should not be cited or distributed as the published version of this manuscript.

BMJ Open is an open access journal and the full, final, typeset and author-corrected version of record of the manuscript is available on our site with no access controls, subscription charges or pay-per-view fees (http://bmjopen.bmj.com).

If you have any questions on BMJ Open's open peer review process please email info.bmjopen@bmj.com

BMJ Open

Violence experience by perpetrator and associations with HIV/STI risk and infection: a cross-sectional study among female sex workers in Karnataka, south India

Journal:	*BMJ Open*
Manuscript ID	bmjopen-2017-021389
Article Type:	Research
Date Submitted by the Author:	26-Dec-2017

Complete List of Authors:	Beksinska, Alicja; London School of Hygiene and Tropical Medicine Department of Global Health and Development,<br>Prakash, Ravi; Karnataka Health Promotion Trust<br>Isac, Shajy; Karnataka Health Promotion Trust<br>Mohan, H L; Karnataka Health Promotion Trust<br>Platt, Lucy; London School of Hygiene and Tropical Medicine Faculty of Public Health and Policy, Department of Social and Environmental Health<br>Blanchard, James; University of Manitoba, Department of Community Health Sciences; University of Manitoba, Department of Medical Microbiology<br>Moses, Stephen; University of Manitoba, Community Health Sciences; University of Manitoba, Department of Medical Microbiology<br>Beattie, Tara; London School of Hygiene and Tropical Medicine Department of Global Health and Development

Keywords:	HIV & AIDS < INFECTIOUS DISEASES, Female Sex Workers, Sexually transmitted infections, Violence

SCHOLARONE™
Manuscripts

VIOLENCE EXPERIENCE BY PERPETRATOR AND ASSOCIATIONS WITH HIV/STI RISK AND INFECTION: A CROSS-SECTIONAL STUDY AMONG FEMALE SEX WORKERS IN KARNATAKA, SOUTH INDIA

Alicja Beksinska[1], Ravi Prakash[2], Shajy Isac[2], H.L. Mohan[2], Lucy Platt[3], James Blanchard[4,5], Stephen Moses[4,5], Tara S Beattie[1]

Institution(s): [1]London School of Hygiene and Tropical Medicine, Department of Global Health and Development, London, United Kingdom, [2]Karnataka Health Promotion Trust, Bengaluru, India, [3]London School of Hygiene and Tropical Medicine, Department of Social and Environmental Health, Faculty of Public Health and Policy, London, United Kingdom, [4]University of Manitoba, Department of Community Health Sciences, Winnipeg, Canada, [5]University of Manitoba, Department of Medical Microbiology, Winnipeg, Canada

Corresponding author:
Alicja Beksinska
Department of Global Health and Development, London School of Hygiene and Tropical Medicine, 15-17 Tavistock Place, London WC1H 9SN, UK
Email address: A.Beksinska1@uni.bsms.ac.uk
Tel no.: 07796980683

Word count: 3157

For peer review only

ABSTRACT

Objectives

Female sex workers (FSWs) experience violence from perpetrators in domestic, workplace and community environments, but little is known about how violence experience by perpetrator and across settings impacts on HIV/STI risk. We examined whether HIV/STI risk differs by the perpetrator of violence, and the environment in which it occurs (domestic and workplace settings).

Methods

An Integrated Biological and Behavioural Assessment (IBBA) survey was conducted among random samples of FSWs in two districts (Bangalore and Shimoga) in Karnataka state, south India, in 2011. Physical and sexual violence in the past 6 months, by workplace (client, police, co-worker, pimp) or community (stranger, rowdy, neighbour, auto-driver) perpetrators was assessed, as was physical and sexual intimate partner violence in the past 12 months. Weighted, bivariate and multivariate analyses were used to examine associations between violence by perpetrator and HIV/STI risk.

Results

1111 FSWs were included (Bangalore=718, Shimoga=393). Overall, 34.9% reported recent physical and/or sexual violence. Violence was experienced from domestic (27.1%), workplace (11.1%) and community (4.2%) perpetrators, with 6.2% of participants reporting recent violence from both domestic *and* non-domestic (workplace/community) perpetrators. Adjusted analysis suggests that experience of workplace/community violence is more important in increasing HIV/STI risk during sex work (lower condom use with clients; client or FSW under the influence of alcohol at last sex) than domestic violence. However, women who reported recent domestic *and* workplace/community violence had the highest odds of high-titre syphilis infection, recent STI symptoms and condom breakage at last sex, and the lowest odds of condom use at last sex with regular clients, compared with women who reported violence by domestic or workplace/community perpetrators only.

For peer review only

Conclusion

HIV/STI risk differs by the perpetrator of violence and is highest among FSWs experiencing violence in the workplace/community *and* at home. Effective HIV/STI prevention programmes with key populations such as FSWs need to include violence interventions that address violence across both their personal and working lives.

Strengths and limitations

- This study is the first to examine the association between violence exposure from multiple perpetrators and HIV/STI risk and prevalence among FSWs.
- The study used a robust sampling strategy and had a reasonably large sample size (>1000 FSWs).
- Intimate partner violence (IPV) was assessed using the validated 8-item WHO IPV questionnaire. However, violence experience by other perpetrators was assessed used a 2-item question which may have led to under-reporting of workplace/community violence.
- The categorisation of violence by perpetrators was based on crude definitions, which likely do not reflect the fluidity of relationships (for example client to intimate partner and vice versa).
- Some associations may have been due to chance, particularly for outcomes with small numbers, such as syphilis infection.

For peer review only - http://bmjopen.bmj.com/site/about/guidelines.xhtml

INTRODUCTION

Violence, in particular, gender-based violence, is recognised as a risk factor for HIV and sexually transmitted infections (STIs)[1]. Female sex workers (FSWs) experience high levels of violence and HIV/STIs[2]. Recent estimates indicate FSWs have a lifetime violence prevalence of 41%-65%[3] compared to 27.8%-32.2%[4] amongst women in the general population as well as 13.5 (10.0-18.1) times the odds of HIV infection[5]. FSWs commonly experience violence on entry into sex work when they are at their most vulnerable[2]. FSWs can experience violence in their workplace from a range of perpetrators including police, clients, pimps and madams[6-10], as well as in their community from private militias, religious groups and others who may perceive sex workers to be 'immoral' and blame them for the spread of HIV and STIs[6]. FSWs may also experience high levels of domestic violence, from intimate partners[11 12], which may be as important as workplace violence in contributing to HIV/STI risk[13 14]. However, little is known about how violence experience by perpetrator and across environments impacts on HIV/STI risk behaviours.

Violence against FSWs is associated with increased HIV/STIs[7 8] and STI symptoms[7 15], and can hinder HIV prevention programming[11]. Recent violence experience may directly increase HIV/STI risk through condom breakage/failure or condom non-use[16-18]. Furthermore, men who perpetrate violence against women are more likely to engage in high risk behaviours including having multiple sexual partners, high alcohol consumption and inconsistent condom use, and have an increased prevalence of HIV, STIs and STI symptoms. This puts their sexual partners at increased HIV/STI risk[2]. HIV vulnerability may be increased indirectly as fear of police violence or arrest may result in women not carrying condoms or working in more isolated, dangerous locations[19], and deter them from accessing sexual health services[6]. Alcohol use is common among FSW populations[20] and their clients and is associated with increased HIV/STI risk[21] and violence experience[22 23].

India has the third largest HIV epidemic globally, with prevalence rates among FSWs ranging from 2-38%. Karnataka state in south India has one of the highest HIV burdens among FSWs, with prevalence previously reaching >30% in some districts[7]. Although sex work per se is not illegal, many FSWs and police wrongly understood this to be the case and sex work is highly stigmatised[23 24]. Violence against FSWs has been identified as a key concern[8]. In 2003, the Karnataka Health Promotion Trust (KHPT), in partnership with the University of Manitoba, was established to scale up HIV prevention programming with 'high-risk' populations. At scale, the

For peer review only - http://bmjopen.bmj.com/site/about/guidelines.xhtml

intervention worked with over 60,000 FSWs per annum using a rights-based approach to address violence, stigma, and poverty as part of comprehensive HIV prevention programming[24]. Changes in behaviour and HIV and STI prevalence were assessed using serial Integrated Biological and Behavioural Assessment (IBBA) cross-sectional surveys.

This study examines whether HIV/STI risk differs depending on the perpetrator of violence, and if this risk is highest among FSWs experiencing violence across multiple environments (e.g. domestic *and* workplace).

For peer review only

METHODS

Study Design

Data were collected from two districts (Shimoga and Bangalore) in the third round of a series of IBBA surveys, in Karnataka state. Intervention programmes were first implemented in 2004. Round 3 IBBA surveys took place in July and August 2011[7].

Sample size calculations have been reported previously[8]. In brief, the target sample for each IBBA district was fixed at 400. To represent the greater number of FSWs in Bangalore and the variation in sex work typology, a sample size of 800 was used [8 25]. Following mapping of FSWs across the two districts, two different sampling methods were used. For FSWs working at brothels, lodges, homes, and *dhabas* (road-side eating establishments) with a more fixed population, a conventional cluster sampling method was used. For street-based FSWs, time-location cluster sampling was utilised. Inclusion criteria were women aged 18-49 years who had received money or gifts in exchange for sex at least once in the past month. FSWs gave written or witnessed verbal informed consent and were interviewed by trained female interviewers in a rented room close to their workplace[8 25 26]. No identifying information was recorded.

The behavioural questionnaire was initially prepared in English and then translated into the local language, Kannada. It included one question on non-partner physical violence [*"In the last six months, how many times would you say someone has beaten you? (hurt, hit, slapped, pushed, kicked, punched, choked, burned?) Who did this to you?"]* and one question on non-partner sexual violence *("in the past one year, has anyone besides your main partner ever forced you to have sexual intercourse when you did not want to? If yes, who was/were this/these person/s?")*[7]. In round 3 in Bangalore and Shimoga, detailed questions on physical (6 items) and sexual violence from non-paying intimate partners (2 items) in the last 12 months were also included based on WHO operational definitions of violence[27] (Appendix A).

Laboratory Methods

Blood samples were taken to test for HIV and syphilis. A confirmed syphilis infection was defined by having a Rapid Plasma Reagin (RPR) positive and a Treponema Pallidum Haemagglutination Assay (TPHA) positive test with an RPR titre of greater than 1:8 classified as high-titre syphilis. Further details of laboratory methods have been previously reported[26].

For peer review only - http://bmjopen.bmj.com/site/about/guidelines.xhtml

Statistical Analyses

The analysis was carried out in STATA 13.1. To take account of sampling probabilities at district, primary sampling unit, and individual levels, as well as rates of non-response, data were appropriately weighted. The main exposure, violence, was categorised into workplace perpetrators (clients, police, pimps, madams and co-workers); community perpetrators (strangers, rowdies, neighbours, auto drivers, assistant ward boys, friends and relatives); and domestic perpetrators (husbands, regular partners and lovers). The primary outcomes were HIV, syphilis and STI symptom prevalence. Secondary outcomes included condom use at last sex; condom breakage at last sex; client or FSW under the influence of alcohol during last sex; STI clinic visit in the past 6 months; and contact with a peer educator in the past month. Associations were measured using odds ratios (ORs) and the Wald chi-square test. For multivariate analysis, age and district were selected as *a priori* confounders. Confounders were identified separately for each outcome using a change-in-estimate approach, but to increase the uniformity of the multivariate models, all outcomes were finally adjusted for the same variables. The adjusted Wald test was used to test for effect modification.

Ethical Considerations

This study was approved by the ethical review board of St Johns Medical College in Bangalore, India (IRB: 179/2010); the Research Ethics Board at the University of Manitoba, Canada (IRB: H2005:098); and the Research Ethics Committee at the London School of Hygiene and Tropical Medicine (IRB: 11118).

For peer review only

RESULTS

Study Population and Violence Experience

Overall, 1111 FSWs participated in the study [Shimoga (n=393), Bangalore (n=718]. Over one-third (34.9%) of FSWs reported recent physical and/or sexual violence with recent physical violence (29.6%) more prevalent than recent sexual violence (21.9%) (Table 1). Reported domestic violence experience was high, with sixty percent of FSWs reporting intimate partner violence (IPV) in their lifetime and over a quarter of women (27.1%) reporting recent domestic violence (past 12 months). Recent workplace violence (past 6 months) was reported by 11.1% of FSWs, with sexual violence (8.2%) more prevalent than physical violence (5.4%). Workplace violence was mainly perpetrated by clients (9.2%), with <1% perpetrated by police, co-workers, and pimps. Recent violence by perpetrators from the community (past 6 months) was the least prevalent (4.1%) and was perpetrated mainly by strangers (2.1%) and 'rowdies' (1.1%) (Table 1).

Due to the small number of women who reported community violence, for the remaining analyses, workplace and community violence were combined into one category. 6.2% of women reported recent violence experience from both domestic *and* non-domestic (workplace or community) perpetrators (Fig. 1).

Table 1 Physical and sexual violence by perpetrator

Type of violence, by perpetrator		Recent Physical Violence %	Recent Sexual Violence[1] %	Recent physical and/or sexual violence %
Overall		29.6	21.9	34.9
Recent domestic violence		25.1	14.7	27.1
	Husband/regular partner	25.1	14.7	27.1
Recent workplace violence		5.4	8.2	11.1
	Client	4.0	7.2	9.2
	Police	0.5	0.9	0.9
	Co-worker	1.0	0.0	1.0
	Pimp	0.0	0.2	0.2
Recent community violence		2.7	2.9	4.2
	Strangers	1.6	1.1	2.1
	Rowdies	0.7	1.0	1.1
	Neighbours	0.3	0.0	0.3
	Auto driver	0.1	0.0	0.1

For peer review only - http://bmjopen.bmj.com/site/about/guidelines.xhtml

Assistant ward boy	0.0	0.05	0.1
Relatives	0.2	0.4	0.5
Friends	0.0	0.4	0.4

Missing observations: [1] n=19 (1.7%)

The mean age of respondents was 32.9 years, and 54.5% were illiterate (Table 2). Two-thirds (66.2%) had a regular partner, and the majority of women had at least one child. Two-thirds (66.1%) had an additional income to sex work. Women solicited clients either by phone (56.7%) or from public places (32.5%). The median number of clients entertained per week was 6 [range 1-70; interquartile range (IQR) 4, 10] and 15.6% had ever practiced sex work outside the district.

Amongst FSWs who experienced recent violence, socio-demographic and sex work characteristics differed by the perpetrator of violence (Table 2). Women who reported recent workplace/community violence were more likely to solicit clients from public places (53.7%), whereas women who reported recent domestic violence only were more likely to solicit clients by phone (53.9%). A higher median number of clients per week was reported among women who experienced workplace/community violence (9; IQR: 5-12) or violence in both domestic *and* workplace/community environments (9; IQR: 6-15), and these women were more likely to have migrated for sex work compared with women who had experienced domestic violence only or no violence (Table 2). Women who reported recent domestic *and* workplace/community violence had the lowest mean age at start of sex work (25.4 years) and lowest mean age at first sex (15.4 years).

Table 2 Socio-demographic and sex work characteristics of FSWs in Shimoga and Bangalore and associations with violence by perpetrator

**Characteristic**		**Overall**	**Recent violence by perpetrator**				
			**No violence**	**Domestic violence only**	**Workplace and/or community violence only**	**Domestic and workplace/ community violence**	**P value (chi square test)**
			%(n=727)	*%(n=216)*	*%(n=80)*	*%(n=69)*	
Age, years	<25	13.1	12.2	13.3	22.0	12.7	0.18
	25-29	22.4	20.9	24.6	21.9	36.1	
	30-39	45.2	44.6	47.4	41.0	42.7	
	40+	19.3	22.4	14.6	15.1	8.5	
	Mean	32.9	33.4	32.2	31.1	30.8	

For peer review only - http://bmjopen.bmj.com/site/about/guidelines.xhtml

Literacy	Illiterate	54.5	56.2	56.2	37.8	57.9	0.07
Marital Status	Lives alone	44.2	53.3	8.3	78.7	18.1	<0.0001
	Lives with partner other than husband	4.5	4.4	4.6	5.8	4.6	
	Married and lives with husband	51.2	42.3	87.0	15.5	76.2	
Regular partner	Yes	66.2	58.3	95.1	46.6	88.2	<0.0001
Number of children	0	9.7	8.7	7.3	22.5	12.2	0.03
	1-2	60.3	62.0	60.1	57.8	53.9	
	3+	30.1	29.4	32.6	19.7	34.0	
	Mean	2.0	2.0	2.2	1.7	2.0	
District	Bangalore	50.8	54.0	42.1	64.1	50.2	0.022
	Shimoga	49.3	46.0	57.9	35.9	49.8	
Additional income to sex work[1]	Yes	66.1	67.5	67.7	58.5	59.5	0.31
Age at first sex (years)	<15	48.2	49.4	44.9	38.9	64.6	0.07
	15+	51.8	50.6	55.1	61.1	35.4	
	Mean	16.1	16.1	16.3	16.6	15.4	
Age started sex work (years)	<20	5.5	5.6	4.8	8.2	5.7	0.056
	20-24	25.2	22.5	26.8	31.1	37.6	
	25-29	29.0	26.8	33.5	30.9	32.5	
	30+	40.3	45.1	35.0	29.8	24.3	
	Mean	28.3	28.9	27.7	26.9	25.4	
Place of solicitation of sex work	Home	7.4	8.4	7.9	3.1	4.7	0.0008
	Rented room/lodge/brothel	3.4	2.6	6.7	0.6	2.4	
	Public place/tamasha/other	32.5	28.3	31.5	53.7	43.4	
	Phone	56.7	60.7	53.9	42.6	49.4	
How much charged for sex with last client (rupees)	400+	53.1	53.1	52.2	62.6	46.7	0.43
	Mean	459.3	469.8	442.5	458.5	422.3	
Number of clients/ week	1-4	28.4	28.2	34.4	18.2	12.8	<0.0001
	5-9	45.0	46.6	47.3	37.7	42.0	
	10+	26.6	25.2	18.3	44.1	45.2	
	Median	6.0	6.0	6.0	9.0	9.0	
Migrant sex work (ever practiced sex work outside the district and/or in Mumbai)	Yes	15.6	12.6	11.9	39.9	25.8	<0.0001

Missing observations: [1] n=6(0.5%), [2] n=1(0.1%)

For peer review only - http://bmjopen.bmj.com/site/about/guidelines.xhtml

For peer review only

HIV/STI risk

Overall HIV prevalence was 8.2%, reactive syphilis 3.1% and high-titre syphilis 0.5%. In multivariate analysis, there was no evidence of an association between violence by perpetrator and either HIV (P-value: 0.27) or reactive syphilis (P-value: 0.76) (Table 3). However, there was strong evidence (P-value <0.0001) for an increased odds of high-titre syphilis infection amongst women who reported recent violence in both their domestic *and* workplace/community environments compared with women who reported no recent violence (aOR: 24.96; 95% CI: 5.94-96.70).

Self-report of STI symptoms (vaginal discharge/genital ulcers/abdominal pain not associated with menses) in the past year was higher amongst women who reported recent violence compared to FSWs who reported no violence. In multivariate analyses, there was strong evidence for an increased odds of STI symptoms in all categories of violence by perpetrator, with those who experienced both domestic *and* workplace/community violence having the highest odds of STI symptoms (aOR: 3.90; 95% CI: 2.10-7.26) (Table 3).

Condom Use

Recent violence by a specific perpetrator was associated with reduced condom use in that setting (Table 3). In adjusted analyses, any recent violence experience, regardless of the perpetrator, was associated with a significant reduction in reported condom use at last sex with occasional and regular clients. In multivariate analysis, recent violence experience by workplace perpetrators, or by domestic *and* workplace/community perpetrators, was significantly associated with reduced condom use with last occasional client and last regular client, compared with women reporting no recent violence. Overall, just one-fifth (19.5%) of FSWs reported condom use at last sex with a regular partner. Reported condom use with regular partners was lower amongst women reporting recent domestic violence compared with women reporting no recent domestic violence, although this association did not remain significant in multivariate analyses.

Condom breakage at last sex was more likely among women who reported any recent violence (5.1%) compared to those who did not report recent violence (1.2%). In multivariate analysis there was strong evidence (P-value: 0.0001) for increased condom breakage among women who reported recent domestic violence (aOR: 3.72;

For peer review only - http://bmjopen.bmj.com/site/about/guidelines.xhtml

95%CI: 1.13-12.25), with the highest odds amongst women who reported both domestic *and* workplace/community violence (aOR: 19.29; 95%CI: 5.42-68.73).

Alcohol use

In univariate and adjusted analyses women who reported recent workplace/community violence (53.9%; aOR: 1.66; 95%CI: 0.96-2.84; P value: 0.024) and both domestic *and* workplace/community violence (56.0%; aOR: 2.16; 95%CI: 1.19-3.92, P-value: 0.024) were more likely to report either themselves, their client or both being under the influence of alcohol at last sex compared to women who reported no violence or domestic violence only.

Programme Exposure

Women who reported any recent violence were more likely to have visited an STI clinic in the last six months (44.5%) compared to those who did not report recent violence (27.8%) with the highest aOR amongst those who reported both recent domestic *and* workplace/community violence (aOR: 3.18; 95% CI: 1.68-6.03). Women who had experienced any recent violence (96.9%) were more likely to have had contact with a peer educator in the past month compared to women who had not experienced recent violence (92.0%), with some evidence for this association in multivariate analyses (aOR: 2.22; 95% CI: 0.98-5.00; P-value: 0.055).

For peer review only

Table 3 Violence by perpetrator and associations with HIV/STI prevalence and sexual risk behaviours

		Recent violence from any perpetrator			Violence by perpetrator (reference group: no recent violence)			
		**No recent violence** *%(n=727)*	**Any recent violence** *%(n=365)*	**P value**	**Domestic violence only** *%(n=216)*	**Workplace and/or community violence only** *%(n=80)*	**Domestic *and* workplace or community violence** *%(n=69)*	**P value***
HIV	%	8.1	6.1		2.5	13.4	8.9	
	Crude OR	1.0	0.73(0.42-1.27)	0.26	0.28(0.11-0.73)	1.75(0.88-3.5.0)	1.11(0.40-3.38)	0.022
	Adjusted OR	1.0	0.82(0.44-1.53)	0.53	0.40(0.15-1.09)	1.16(0.55-2.44)	1.32(0.41-4.29)	0.27
Reactive syphilis[1]	%	3.4	2.9		1.5	6.2	3.5	
	Crude OR	1.0	0.87(0.40-1.91)	0.74	0.42(0.09-2.02)	2.04(0.60-6.89)	1.12(0.30-4.22)	0.60
	Adjusted OR	1.0	1.27(0.68-2.38)	0.46	1.14(0.30-4.46)	1.17(0.57-2.40)	2.04(0.53-7.81)	0.76
High titre syphilis	%	0.38	0.64		0	0.9	2.5	
	Crude OR	1.0	1.70(0.36-8.03)	0.50	-	2.36(0.25-22.07)	6.74(1.15-39.58)	0.11
	Adjusted OR	1.0	2.22(0.54-9.17)	0.27	-	2.27(0.26-19.8)	24.96(5.94-96.70)	<0.0001
STI symptoms in past 12 months (vaginal discharge, lower abdominal pain not associated with menses and/or genital ulcer)	%	30.7	48.9		41.5	57.3	63.3	
	Crude OR	1.0	2.16(1.61-2.89)	<0.0001	1.60(1.11-2.31)	3.03(1.77-5.18)	3.90(2.18-6.95)	<0.0001
	Adjusted OR	1.0	2.27(1.66-3.09)	<0.0001	1.87(1.24-2.81)	2.41(1.40-4.17)	3.90(2.10-7.26)	<0.0001
Condom use last sex with occasional client[2]	%	97.5	94.5		97.2	91.6	91.0	
	Crude OR	1.0	0.45(0.21-0.96)	0.038	0.87(0.29-2.63)	0.28(0.10-0.74)	0.26(0.09-0.75)	0.0073
	Adjusted OR	1.0	0.39(0.19-0.83)	0.014	1.03(0.33-3.28)	0.20(0.07-0.52)	0.22(0.06-0.81)	0.0001

For peer review only

For peer review only - http://bmjopen.bmj.com/site/about/guidelines.xhtml

Condom use last sex with regular client[3]	%	93.0	88.2		92.6	85.9	76.0	
	Crude OR	1.0	0.56(0.32-0.98)	0.043	0.94(0.44-2.01)	0.46(0.20-1.05)	0.24(0.11-0.50)	0.0012
	Adjusted OR	1.0	0.61(0.32-1.15)	0.12	1.25(0.54-2.90)	0.33(0.15-0.73)	0.25(0.10-0.59)	0.0003
Condom use at last sex with regular partner	%	23.1	13.8		12.2	27.8	10.4	
	Crude OR	1.0	0.53(0.32-0.89)	0.016	0.46(0.26-0.84)	1.29(0.51-3.22)	0.39(0.16-0.93)	0.012
	Adjusted OR	1.0	0.63(0.35-1.14)	0.13	0.79(0.42-1.51)	0.40(0.14-1.12)	0.48(0.14-1.67)	0.30
Condom breakage at last sex[4]	%	1.2	5.1		3.0	3.1	15.1	
	Crude OR	1.0	4.38(1.91-10.02)	0.0005	2.46(0.84-7.25)	2.60(0.62-10.9)	14.3(5.10-40.30)	<0.0001
	Adjusted OR	1.0	4.32(1.74-10.73)	0.0017	3.72(1.13-12.25)	1.71(0.36-8.20)	19.29(5.42-68.73)	0.0001
Either client, FSW or both under the influence of alcohol at last sex	%	35.8	42.4		34.1	53.9	56.0	
	Crude OR	1.0	1.32(0.99-1.76)	0.058	0.93(0.661.30)	2.09(1.24-3.52)	2.28(1.31-3.99)	0.0015
	Adjusted OR	1.0	1.29(0.09-1.77)	0.12	0.97(0.66-1.42)	1.66(0.96-2.84)	2.16(1.19-3.92)	0.024
Visited an STI clinic in past six months for STI symptoms[5]	%	27.8	44.5		39.0	51.6	53.2	
	Crude OR	1.0	2.08(1.49-2.92)	<0.0001	1.66(1.10-2.52)	2.77(1.61-4.78)	2.95(1.61-5.43)	<0.0001
	Adjusted OR	1.0	2.28(1.59-3.27)	<0.0001	2.04(1.28-3.24)	2.32(1.35-3.97)	3.18(1.68-6.03)	0.0001
Had contact with a peer educator in the last month[6]	%	92.0	96.9		96.3	100	95.2	
	Crude OR	1.0	2.74(1.24-6.07)	0.013	2.27(0.86-6.00)	-	1.73(0.55-5.43)	0.20
	Adjusted OR	1.0	2.22(0.98-5.00)	0.055	1.75(0.63-4.90)	-	1.18(0.36-3.92)	0.56

Models adjusted for age, district, marital status, migrant sex work, place of selling sex and having an income other than sex work;
Missing observations: [1] n=1 (0.1%), [2] n=1(0.1%), [3] n=1(0.1%), [4] n=3(0.3%), [5] n=159 (14.3%), [6] n=22(2.0%); *Wald test: tests the null hypothesis that the coefficients of interest are simultaneously equal to zero

For peer review only

For peer review only - http://bmjopen.bmj.com/site/about/guidelines.xhtml

DISCUSSION

We found a high prevalence of violence from a range of perpetrators experienced by FSWs in this setting in India, with recent domestic violence more commonly reported than workplace or community violence. Additionally, we found that HIV/STI risk differed by perpetrator of violence and was highest amongst women who reported recent violence across multiple environments; women reporting recent domestic *and* workplace/community violence were significantly more likely to have high-titre syphilis infection and had the highest odds of recent STI symptoms, condom breakage at last sex, alcohol use at last sex and no condom use at last sex with regular clients. This study is the first of its kind to show that increased STI prevalence and HIV/STI risk among FSWs is associated with experience of violence across multiple environments. It also adds to a growing body of research globally, reporting the burden and range of perpetrators of violence among FSWs. In South Africa, HIV/STI risks among women from the general population have been found to be highest amongst those experiencing the highest levels of violence[28 29], suggesting a 'dose-response' effect between violence and HIV/STI risk. This may partly explain the increased STI prevalence and sexual risk behaviours amongst women in our study who reported violence from multiple perpetrators.

Despite the high rates of domestic violence, our study findings suggest that workplace/community violence is more important for increasing sexual risk behaviours overall and during sex work, compared with domestic violence. Previous studies with FSWs in India have reported conflicting associations between IPV and condom use with clients[30 31]. In our study, we found no associations between domestic violence and sexual risk behaviours in the workplace, such as condom use with clients. However, women in our study who reported domestic violence only did have increased odds of STI symptoms and condom breakage at last sex compared to women who did not report recent violence, suggesting that domestic violence is associated with some level of increased HIV/STI risk. A recent systematic review of domestic violence among women in India estimated the median prevalence of lifetime and domestic violence in the past year was 41% and 30%, respectively[32]. In our study FSWs experienced an even higher prevalence of lifetime (60.1%) and recent domestic violence (27.1%). These high levels of domestic violence need to be addressed to reduce impacts on physical and psychological health[33]. So far, HIV prevention programmes with FSWs have focused mainly on reducing workplace violence[7 8 23] and improving condom use with clients[25]. Although there are examples

For peer review only - http://bmjopen.bmj.com/site/about/guidelines.xhtml

of successful interventions to reduce domestic violence in women in the general population[34], the efficacy of such interventions among FSWs is unknown. A cluster RCT with FSWs, in Karnataka India, aimed at reducing IPV and improving condom use with their lover/husband is currently being assessed, and is the first of its kind to address domestic violence among FSWs[35].

Prevalence of recent workplace violence was high (11.1%) despite the success of recent violence interventions in Karnataka[7], with clients the major perpetrators. Reported community violence was low (4.1%) compared to violence from other perpetrators. However, FSWs may be at greater risk of violence from community perpetrators compared to women in the general population, due to stigma and dangerous working environments. An important finding was the strong association between having experienced both domestic *and* workplace/community violence and increased odds of high-titre syphilis, demonstrating biological evidence of increased STI risk. As high-titre syphilis infection indicates recent infection, the direction of the association is more plausible compared to measures of chronic STI infection (HIV and reactive syphilis). Unfortunately in IBBA R3, FSWs were not tested for other incident STIs, due to budget constraints, although violence has been associated with gonorrhea in previous IBBAs[7]. Self-reported STI symptoms were strongly associated with violence from all perpetrators with the highest odds amongst those who reported both domestic *and* workplace/community violence. Although this may indicate STI infection in some cases, self-reported STI symptoms are not a reliable indicator of biological infection[36]. Vaginal discharge in women in India has been linked to depression and psychosocial stress, which may partly explain this association[37]. The reduced odds of condom use with clients amongst women who reported workplace/community violence and both domestic *and* workplace/community violence, but not domestic violence, indicates that the association between violence and HIV/STI risk may be driven by the environment in which the violence occurs.

The finding that FSWs who report recent violence have higher STI clinic attendance and recent contact with a peer educator reflects positively on the HIV/STI prevention programme in Karnataka, suggesting recent experience of violence does not hinder women from accessing services. In this study, having experienced workplace/community violence and both domestic *and* workplace/community violence was associated with alcohol use at last sex. Having experienced violence can lead to increased alcohol consumption as a coping mechanism[21 23]. Alternatively

For peer review only - http://bmjopen.bmj.com/site/about/guidelines.xhtml

being under the influence of alcohol may increase vulnerability to violence and arrest[21].

This study had strengths and limitations. Although previous research has examined IPV and workplace violence among FSWs[31], none have included community violence or examined associations with biological STI infection. Only one previous study in Soweto, South Africa has reported on the prevalence of violence experience across multiple environments among FSWs[10], but this study did not examine associations with HIV/STI risk. To our knowledge, our study is the first to demonstrate increased prevalence of STI infection and sexual risk behaviours among FSWs who experience violence across multiple environments, compared with FSWs who report either no recent violence, or recent violence in a domestic or workplace setting only. Other important strengths were the robust sampling strategy and the reasonably large sample size. With cross-sectional data, it is not possible to ascertain the direction of association for some outcomes or infer causality. Reporting bias may have contributed to over-reporting of certain outcomes (such as condom use) while more stigmatized and sensitive topics (such as alcohol consumption and violence) may have been under-reported. The categorisation of violence by perpetrators was based on crude definitions, which likely do not reflect the fluidity of relationships. For example, women who sell sex at home may experience domestic and workplace violence in one physical environment while the definition between regular client and lover/partner can become blurred, with clients becoming lovers and vice versa. Some associations may have been due to chance, particularly for outcomes with small numbers and wide confidence intervals, such as high titre syphilis infection. If the WHO standardized 13-item violence questionnaire, which has been shown to yield higher response rates[8], had also been used for non-partners, it might have increased reporting of violence from workplace and community perpetrators.

For peer review only

Despite these limitations, the findings of this study have important implications for HIV/STI prevention among FSWs. Violence against FSWs across both domestic, workplace and community settings needs to be addressed through integrated, comprehensive HIV programmes to enforce their human right to be able to live and work without fear for their safety.

For peer review only - http://bmjopen.bmj.com/site/about/guidelines.xhtml

Contributors:

AB conducted the analyses, and wrote the first draft of the manuscript. RP supervised the analyses and reviewed the article. RP, SI, HLM, LP, JB, and SM contributed to the study design and reviewed the article. TSB conceptualised the study, supervised the analyses, and reviewed the article.

Funding:

This study was supported by the India AIDS Initiative (*Avahan*) of the Bill & Melinda Gates Foundation, grant no. OPP52138. It was also funded by the UK Department for International Development (DFID) as part of STRIVE, a 6-year programme of research and action devoted to tackling the structural drivers of HIV (http://STRIVE.lshtm.ac.uk/). Tara Beattie is supported by a British Academy Fellowship. The views expressed herein are those of the authors and do not necessarily reflect the official policy or position of the Bill and Melinda Gates Foundation, UK DFID or the British Academy.

Competing Interests:

None

Data Sharing Statement:

No additional data are available.

For peer review only

REFERENCES

1. Gupta GR, Parkhurst JO, Ogden JA, et al. Structural approaches to HIV prevention. *Lancet* 2008;372(9640):764-75. doi: 10.1016/s0140-6736(08)60887-9 [published Online First: 2008/08/09]
2. Dunkle KL, Decker MR. Gender-based violence and HIV: reviewing the evidence for links and causal pathways in the general population and high-risk groups. *Am J Reprod Immunol* 2013;69 Suppl 1:20-6. doi: 10.1111/aji.12039
3. Deering KN, Amin A, Shoveller J, et al. A systematic review of the correlates of violence against sex workers. *Am J Public Health* 2014;104(5):e42-54. doi: 10.2105/ajph.2014.301909 [published Online First: 2014/03/15]
4. Devries KM, Mak JYT, García-Moreno C, et al. The Global Prevalence of Intimate Partner Violence Against Women. *Science* 2013;340(6140):1527-28. doi: 10.1126/science.1240937
5. Baral S, Beyrer C, Muessig K, et al. Burden of HIV among female sex workers in low-income and middle-income countries: a systematic review and meta-analysis. *Lancet Infect Dis* 2012;12(7):538-49. doi: 10.1016/S1473-3099(12)70066-X
6. Decker MR, Crago A-L, Chu SKH, et al. Human rights violations against sex workers: burden and effect on HIV. *The Lancet*;385(9963):186-99. doi: 10.1016/S0140-6736(14)60800-X
7. Beattie TS, Bhattacharjee P, Isac S, et al. Declines in violence and police arrest among female sex workers in Karnataka state, south India, following a comprehensive HIV prevention programme. *J Int AIDS Soc* 2015;18:20079. doi: 10.7448/IAS.18.1.20079
8. Beattie TS, Bhattacharjee P, Ramesh BM, et al. Violence against female sex workers in Karnataka state, south India: impact on health, and reductions in violence following an intervention program. *BMC Public Health* 2010;10:476. doi: 10.1186/1471-2458-10-476
9. Schwitters A, Swaminathan M, Serwadda D, et al. Prevalence of rape and client-initiated gender-based violence among female sex workers: Kampala, Uganda, 2012. *AIDS and behavior* 2015;19 Suppl 1:S68-76. doi: 10.1007/s10461-014-0957-y [published Online First: 2014/11/30]
10. Coetzee J, Gray GE, Jewkes R. Prevalence and patterns of victimization and polyvictimization among female sex workers in Soweto, a South African township: a cross-sectional, respondent-driven sampling study. *Glob Health Action* 2017;10(1):1403815. doi: 10.1080/16549716.2017.1403815 [published Online First: 2017/12/07]
11. Pando MA, Coloccini RS, Reynaga E, et al. Violence as a barrier for HIV prevention among female sex workers in Argentina. *PLoS One* 2013;8(1):e54147. doi: 10.1371/journal.pone.0054147
12. Decker MR, Pearson E, Illangasekare SL, et al. Violence against women in sex work and HIV risk implications differ qualitatively by perpetrator. *BMC Public Health* 2013;13:876.
13. Panchanadeswaran S, Johnson SC, Sivaram S, et al. Intimate partner violence is as important as client violence in increasing street-based female sex workers' vulnerability to HIV in India. *International Journal of Drug Policy*;19(2):106-12.
14. Deering KN, Bhattacharjee P, Bradley J, et al. Condom use within non-commercial partnerships of female sex workers in southern India. *BMC Public Health*;11 Suppl 6:S11.
15. Decker MR, McCauley HL, Phuengsamran D, et al. Violence victimisation, sexual risk and sexually transmitted infection symptoms among female sex workers in Thailand. *Sexually Transmitted Infections* 2010;86(3):236-40.

For peer review only

16. Tounkara FK, Diabate S, Guedou FA, et al. Violence, condom breakage, and HIV infection among female sex workers in Benin, West Africa. *Sexually Transmitted Diseases* 2014;41(5):312-8.
17. Bradley J, Rajaram S, Moses S, et al. Female sex worker client behaviors lead to condom breakage: a prospective telephone-based survey in Bangalore, South India. *AIDS and behavior* 2013;17(2):559-67. doi: 10.1007/s10461-012-0192-3
18. Choi SY, Chen KL, Jiang ZQ. Client-perpetuated violence and condom failure among female sex workers in southwestern China. *Sex Transm Dis* 2008;35(2):141-6. doi: 10.1097/OLQ.0b013e31815407c3
19. Shannon K, Strathdee SA, Shoveller J, et al. Structural and environmental barriers to condom use negotiation with clients among female sex workers: implications for HIV-prevention strategies and policy. *Am J Public Health* 2009;99(4):659-65. doi: 10.2105/ajph.2007.129858 [published Online First: 2009/02/07]
20. Li Q, Li X, Stanton B. Alcohol Use Among Female Sex Workers and Male Clients: An Integrative Review of Global Literature. *Alcohol and Alcoholism (Oxford, Oxfordshire)* 2010;45(2):188-99. doi: 10.1093/alcalc/agp095
21. Mbonye M, Rutakumwa R, Weiss H, et al. Alcohol consumption and high risk sexual behaviour among female sex workers in Uganda. *African journal of AIDS research : AJAR* 2014;13(2):145-51. doi: 10.2989/16085906.2014.927779 [published Online First: 2014/09/02]
22. Chersich MF, Bosire W, King'ola N, et al. Effects of hazardous and harmful alcohol use on HIV incidence and sexual behaviour: a cohort study of Kenyan female sex workers. *Global Health* 2014;10:22. doi: 10.1186/1744-8603-10-22
23. Biradavolu MR, Burris S, George A, et al. Can sex workers regulate police? Learning from an HIV prevention project for sex workers in southern India. *Social science & medicine (1982)* 2009;68(8):1541-7. doi: 10.1016/j.socscimed.2009.01.040
24. Gurnani V, Beattie TS, Bhattacharjee P, et al. An integrated structural intervention to reduce vulnerability to HIV and sexually transmitted infections among female sex workers in Karnataka state, south India. *BMC Public Health* 2011;11:755. doi: 10.1186/1471-2458-11-755
25. Ramesh BM, Beattie TS, Shajy I, et al. Changes in risk behaviours and prevalence of sexually transmitted infections following HIV preventive interventions among female sex workers in five districts in Karnataka state, south India. *Sexually Transmitted Infections* 2010;86 Suppl 1:i17-24.
26. Reza-Paul S, Beattie T, Syed HU, et al. Declines in risk behaviour and sexually transmitted infection prevalence following a community-led HIV preventive intervention among female sex workers in Mysore, India. *AIDS (London, England)* 2008;22 Suppl 5:S91-100. doi: 10.1097/01.aids.0000343767.08197.18 [published Online First: 2009/01/07]
27. World Health Organisation. WHO multi-country study on women's health and domestic violence against women: Initial results on prevalence, health outcomes and women's responses. Geneva, 2005.
28. Jewkes RK, Dunkle K, Nduna M, et al. Intimate partner violence, relationship power inequity, and incidence of HIV infection in young women in South Africa: a cohort study. *Lancet* 2010;376(9734):41-8.
29. Dunkle KL, Jewkes RK, Brown HC, et al. Gender-based violence, relationship power, and risk of HIV infection in women attending antenatal clinics in South Africa. *Lancet* 2004;363(9419):1415-21. doi: 10.1016/s0140-6736(04)16098-4 [published Online First: 2004/05/04]

For peer review only - http://bmjopen.bmj.com/site/about/guidelines.xhtml

30. Deering K, Bhattacharjee P, Mohan HL, et al. Occupational and intimate partner violence and inconsistent condom use with clients among female sex workers in Southern India. *Sexually Transmitted Infections* 2011;87:A66-A67.
31. Reed E, Erausquin JT, Groves AK, et al. Client-perpetrated and husband-perpetrated violence among female sex workers in Andhra Pradesh, India: HIV/STI risk across personal and work contexts. *Sexually Transmitted Infections* 2016 doi: 10.1136/sextrans-2015-052162
32. Kalokhe A, del Rio C, Dunkle K, et al. Domestic violence against women in India: A systematic review of a decade of quantitative studies. *Global Public Health* 2016:1-16. doi: 10.1080/17441692.2015.1119293
33. World Health Organisation, London School of Hygiene & Tropical Medicine, South African Medical Research Council. Global and regional estimates of violence against women: prevalence and health effects of intimate partner violence and non-partner sexual violence, 2013.
34. Pronyk PM, Hargreaves JR, Kim JC, et al. Effect of a structural intervention for the prevention of intimate-partner violence and HIV in rural South Africa: a cluster randomised trial. *Lancet* 2006;368(9551):1973-83. doi: 10.1016/s0140-6736(06)69744-4 [published Online First: 2006/12/05]
35. Beattie TS, Isac S, Bhattacharjee P, et al. Reducing violence and increasing condom use in the intimate partnerships of female sex workers: study protocol for Samvedana Plus, a cluster randomised controlled trial in Karnataka state, south India. *BMC Public Health* 2016;16:660. doi: 10.1186/s12889-016-3356-7
36. Vishwanath S, Talwar V, Prasad R, et al. Syndromic management of vaginal discharge among women in a reproductive health clinic in India. *Sexually Transmitted Infections* 2000;76(4):303-06. doi: 10.1136/sti.76.4.303
37. Patel V, Pednekar S, Weiss H, et al. Why do women complain of vaginal discharge? A population survey of infectious and pyschosocial risk factors in a South Asian community. *Int J Epidemiol* 2005;34(4):853-62. doi: 10.1093/ije/dyi072 [published Online First: 2005/04/19]

FIGURES

Figure 1 Proportional venn diagram showing overlapping of physical and/or sexual violence experiences among FSWs by perpetrator

APPENDICES

See supplementary files for Appendix A (violence questionnaire)

For peer review only

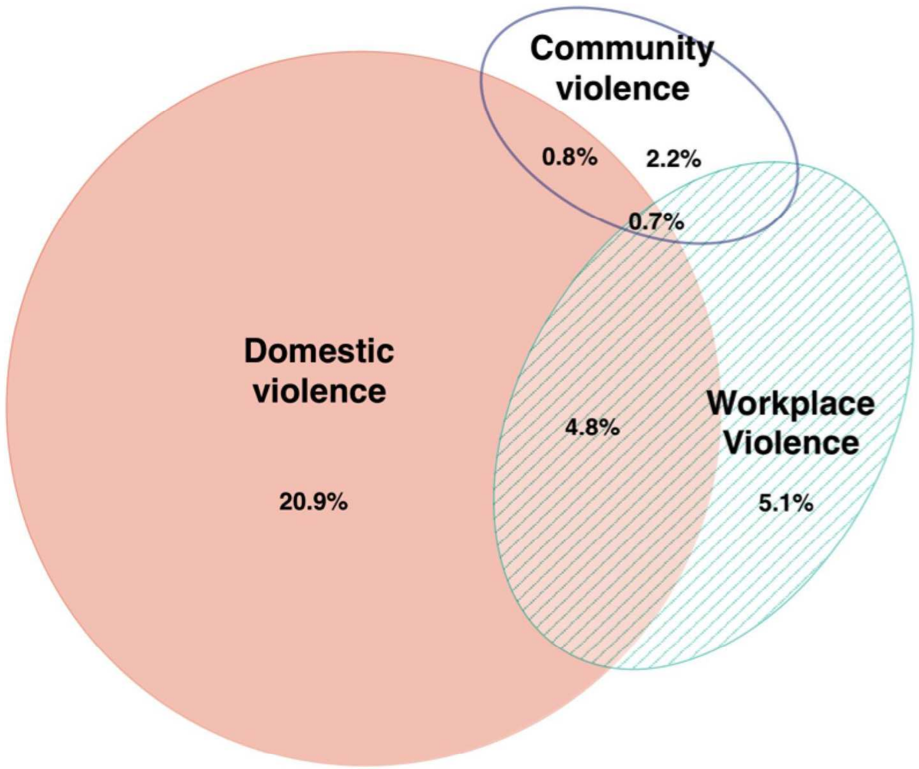

Proportional venn diagram showing overlapping of physical and/or sexual violence experiences among FSWs by perpetrator

146x141mm (300 x 300 DPI)

Appendix A: IBBA violence questionnaire

Physical and sexual violence from a husband/main partner based on WHO operational definitions of violence	Q717b: A. Has any husband or main partner that you have lived with ever done following things to you?<br><br>a) pushed you, shaken you, or thrown something at you?<br><br>b) slapped or shoved you?<br><br>c) hit you with his fist or something else that could hurt you?<br><br>d) kicked you, dragged you or beat you up?<br><br>e) tried to choke you or burn you on purpose<br><br>f) threatened to use or actually used a knife, gun or any other weapon?<br><br>g) physically forced to have sex with him even when you did not want.<br><br>h) used threats of violence or rejection to forced you to have sex with him when you did not want to?<br><br>B. How often has this happened during the last 12 months: often, only sometimes, or not at all?

For peer review only

STROBE checklist for manuscript: Violence experience by perpetrator and associations with HIV/STI risk and infection: a cross-sectional study among female sex workers in Karnataka, south India

STROBE Statement—Checklist of items that should be included in reports of ***cross-sectional studies***

	Item No	Recommendation	Reported on page #
**Title and abstract**	1	(*a*) Indicate the study's design with a commonly used term in the title or the abstract	2
		(*b*) Provide in the abstract an informative and balanced summary of what was done and what was found	2, 3
**Introduction**			
Background/rationale	2	Explain the scientific background and rationale for the investigation being reported	4, 5
Objectives	3	State specific objectives, including any prespecified hypotheses	4,5
**Methods**			
Study design	4	Present key elements of study design early in the paper	5, 6
Setting	5	Describe the setting, locations, and relevant dates, including periods of recruitment, exposure, follow-up, and data collection	6
Participants	6	(*a*) Give the eligibility criteria, and the sources and methods of selection of participants	6
Variables	7	Clearly define all outcomes, exposures, predictors, potential confounders, and effect modifiers. Give diagnostic criteria, if applicable	7
Data sources/ measurement	8*	For each variable of interest, give sources of data and details of methods of assessment (measurement). Describe comparability of assessment methods if there is more than one group	7
Bias	9	Describe any efforts to address potential sources of bias	7
Study size	10	Explain how the study size was arrived at	6
Quantitative variables	11	Explain how quantitative variables were handled in the analyses. If applicable, describe which groupings were chosen and why	7
Statistical methods	12	(*a*) Describe all statistical methods, including those used to control for confounding	7
		(*b*) Describe any methods used to examine subgroups and interactions	7
		(*c*) Explain how missing data were addressed	
		(*d*) If applicable, describe analytical methods taking account of sampling strategy	7
		(*e*) Describe any sensitivity analyses	
**Results**			
Participants	13*	(a) Report numbers of individuals at each stage of study—eg numbers potentially eligible, examined for eligibility, confirmed eligible, included in the study, completing follow-up, and analysed	8
		(b) Give reasons for non-participation at each stage	
		(c) Consider use of a flow diagram	
Descriptive data	14*	(a) Give characteristics of study participants (eg demographic, clinical, social) and information on exposures and potential confounders	9

For peer review only

		(b) Indicate number of participants with missing data for each variable of interest	9, 10, 14
Outcome data	15*	Report numbers of outcome events or summary measures	11, 12, 13, 14
Main results	16	(*a*) Give unadjusted estimates and, if applicable, confounder-adjusted estimates and their precision (eg, 95% confidence interval). Make clear which confounders were adjusted for and why they were included	11, 12, 13, 14
		(*b*) Report category boundaries when continuous variables were categorized	9, 10
		(*c*) If relevant, consider translating estimates of relative risk into absolute risk for a meaningful time period	
Other analyses	17	Report other analyses done—eg analyses of subgroups and interactions, and sensitivity analyses	
**Discussion**			
Key results	18	Summarise key results with reference to study objectives	15, 17
Limitations	19	Discuss limitations of the study, taking into account sources of potential bias or imprecision. Discuss both direction and magnitude of any potential bias	16. 17
Interpretation	20	Give a cautious overall interpretation of results considering objectives, limitations, multiplicity of analyses, results from similar studies, and other relevant evidence	15-17
Generalisability	21	Discuss the generalisability (external validity) of the study results	15, 17
**Other information**			
Funding	22	Give the source of funding and the role of the funders for the present study and, if applicable, for the original study on which the present article is based	18

*Give information separately for exposed and unexposed groups.

Note: An Explanation and Elaboration article discusses each checklist item and gives methodological background and published examples of transparent reporting. The STROBE checklist is best used in conjunction with this article (freely available on the Web sites of PLoS Medicine at http://www.plosmedicine.org/, Annals of Internal Medicine at http://www.annals.org/, and Epidemiology at http://www.epidem.com/). Information on the STROBE Initiative is available at www.strobe-statement.org.

For peer review only

For peer review only - http://bmjopen.bmj.com/site/about/guidelines.xhtml

BMJ Open

Violence experience by perpetrator and associations with HIV/STI risk and infection: a cross-sectional study among female sex workers in Karnataka, south India

Journal:	*BMJ Open*
Manuscript ID	bmjopen-2017-021389.R1
Article Type:	Research
Date Submitted by the Author:	18-Apr-2018
Complete List of Authors:	Beksinska, Alicja; London School of Hygiene and Tropical Medicine Department of Global Health and Development,<br>Prakash, Ravi; Karnataka Health Promotion Trust<br>Isac, Shajy; Karnataka Health Promotion Trust<br>Mohan, H L; Karnataka Health Promotion Trust<br>Platt, Lucy; London School of Hygiene and Tropical Medicine Faculty of Public Health and Policy, Department of Social and Environmental Health<br>Blanchard, James; University of Manitoba, Department of Community Health Sciences; University of Manitoba, Department of Medical Microbiology<br>Moses, Stephen; University of Manitoba, Community Health Sciences; University of Manitoba, Department of Medical Microbiology<br>Beattie, Tara; London School of Hygiene and Tropical Medicine Department of Global Health and Development
**Primary Subject Heading**:	Sexual health
Secondary Subject Heading:	HIV/AIDS, Epidemiology
Keywords:	HIV & AIDS < INFECTIOUS DISEASES, Female Sex Workers, Sexually transmitted infections, Violence

SCHOLARONE™
Manuscripts

VIOLENCE EXPERIENCE BY PERPETRATOR AND ASSOCIATIONS WITH HIV/STI RISK AND INFECTION: A CROSS-SECTIONAL STUDY AMONG FEMALE SEX WORKERS IN KARNATAKA, SOUTH INDIA

Alicja Beksinska[1], Ravi Prakash[2], Shajy Isac[2], H.L. Mohan[2], Lucy Platt[3], James Blanchard[4,5], Stephen Moses[4,5], Tara S Beattie[1]

Institution(s): [1]London School of Hygiene and Tropical Medicine, Department of Global Health and Development, London, United Kingdom, [2]Karnataka Health Promotion Trust, Bengaluru, India, [3]London School of Hygiene and Tropical Medicine, Department of Social and Environmental Health, Faculty of Public Health and Policy, London, United Kingdom, [4]University of Manitoba, Department of Community Health Sciences, Winnipeg, Canada, [5]University of Manitoba, Department of Medical Microbiology, Winnipeg, Canada

Corresponding author:
Alicja Beksinska
Department of Global Health and Development, London School of Hygiene and Tropical Medicine, 15-17 Tavistock Place, London WC1H 9SN, UK
Email address: A.Beksinska1@uni.bsms.ac.uk
Tel no.: 07796980683

Word count: 3998

For peer review only

ABSTRACT

Objectives

Female sex workers (FSWs) experience violence from a range of perpetrators, but little is known about how violence experience across multiple settings (workplace, community, domestic) impacts on HIV/STI risk. We examined whether HIV/STI risk differs by the perpetrator of violence.

Methods

An Integrated Biological and Behavioural Assessment (IBBA) survey was conducted among random samples of FSWs in two districts (Bangalore and Shimoga) in Karnataka state, south India, in 2011. Physical and sexual violence in the past 6 months, by workplace (client, police, co-worker, pimp) or community (stranger, rowdy, neighbour, auto-driver) perpetrators was assessed, as was physical and sexual intimate partner violence in the past 12 months. Weighted, bivariate and multivariate analyses were used to examine associations between violence by perpetrator and HIV/STI risk.

Results

1111 FSWs were included (Bangalore=718, Shimoga=393). Overall, 34.9% reported recent physical and/or sexual violence. Violence was experienced from domestic (27.1%), workplace (11.1%) and community (4.2%) perpetrators, with 6.2% of participants reporting recent violence from both domestic *and* non-domestic (workplace/community) perpetrators. Adjusted analysis suggests that experience of violence by workplace/community perpertrators is more important in increasing HIV/STI risk during sex work (lower condom use with clients; client or FSW under the influence of alcohol at last sex) than domestic violence. However, women who reported recent violence by domestic *and* workplace/community perpetrators had the highest odds of high-titre syphilis infection, recent STI symptoms and condom breakage at last sex, and the lowest odds of condom use at last sex with regular clients, compared with women who reported violence by domestic or workplace/community perpetrators only.

For peer review only - http://bmjopen.bmj.com/site/about/guidelines.xhtml

Conclusion

HIV/STI risk differs by the perpetrator of violence and is highest among FSWs experiencing violence in the workplace/community *and* at home. Effective HIV/STI prevention programmes with FSWs need to include violence interventions that address violence across both their personal and working lives.

Strengths and limitations

- This study is the first to examine the association between violence exposure from multiple perpetrators and HIV/STI risk and prevalence among FSWs.
- The study used a robust sampling strategy and had a reasonably large sample size (>1000 FSWs).
- Intimate partner violence (IPV) was assessed using the validated 8-item WHO IPV questionnaire. However, violence experience by other perpetrators was assessed used a 2-item question which may have led to under-reporting of workplace/community violence.
- The categorisation of violence by perpetrators was based on crude definitions, which likely do not reflect the fluidity of relationships (for example client to intimate partner and vice versa).
- Some associations may have been due to chance, particularly for outcomes with small numbers, such as syphilis infection.

For peer review only - http://bmjopen.bmj.com/site/about/guidelines.xhtml

INTRODUCTION

Violence, in particular, gender-based violence, is recognised as a risk factor for HIV and sexually transmitted infections (STIs)[1]. Female sex workers (FSWs) experience high levels of violence and HIV/STIs[2]. Recent estimates indicate FSWs have a lifetime violence prevalence of 41%-65%[3] compared to 27.8%-32.2%[4] amongst women in the general population as well as 13.5 (10.0-18.1) times the odds of HIV infection[5]. FSWs commonly experience violence on entry into sex work when they are at their most vulnerable[2]. FSWs can experience violence in their workplace from a range of perpetrators including police, clients, pimps and madams[6-10], as well as in their community from private militias, religious groups and others who may perceive sex workers to be 'immoral' and blame them for the spread of HIV and STIs[6]. FSWs also experience high levels of domestic violence, from intimate partners[11 12].

Violence against FSWs is associated with increased HIV/STIs[7 8] and STI symptoms[7 13], and can hinder HIV prevention programming[11]. Recent violence experience may directly increase HIV/STI risk through condom breakage/failure or condom non-use[14-16]. Furthermore, men who perpetrate violence against women are more likely to engage in high risk behaviours including having multiple sexual partners, high alcohol consumption and inconsistent condom use, and have an increased prevalence of HIV, STIs and STI symptoms. This puts their sexual partners at increased HIV/STI risk[2]. HIV vulnerability may be increased indirectly as fear of police violence or arrest may result in women not carrying condoms or working in more isolated, dangerous locations[17], and deter them from accessing sexual health services[6]. Alcohol use is common among FSW populations[18] and their clients and is associated with increased HIV/STI risk[19] and violence experience[20 21].

India has the third largest HIV epidemic globally, with prevalence rates among FSWs ranging from 2-38%. Karnataka state in south India has one of the highest HIV burdens among FSWs, with prevalence previously reaching >30% in some districts[7]. Although sex work per se is not illegal, many FSWs and police wrongly understood this to be the case and sex work is highly stigmatised[21 22]. Violence against FSWs has been identified as a key concern[8]. In 2003, the Karnataka Health Promotion Trust (KHPT), in partnership with the University of Manitoba, was established to scale up HIV prevention programming with 'high-risk' populations. At scale, the intervention worked with over 60,000 FSWs per annum using a rights-based approach to address violence, stigma, and poverty as part of comprehensive HIV prevention programming[22]. Changes in behaviour and HIV and STI prevalence were

For peer review only - http://bmjopen.bmj.com/site/about/guidelines.xhtml

assessed using serial Integrated Biological and Behavioural Assessment (IBBA) cross-sectional surveys.

Studies examining the association between violence and HIV/STI infection and sexual risk behaviours among FSWs have primarily focused on client violence. Although there is now evidence of how HIV prevention programmes among FSWs can effectively reduce violence from non-partners[7 8 21] there has been less research on the impacts of domestic violence on HIV/STI risk, or the efficacy of programmes targeting domestic violence among FSWs. The complexity of violence from different perpetrators and the associated HIV/STI risks are still unclear which hinders the ability of researchers and policy-makers to design violence prevention programmes. Qualitative research has suggested that domestic violence may be as important as workplace violence in contributing to HIV/STI risk[23] and FSWs report low levels of condom use with intimate partners[24]. To our knowledge only one previous study in Andhra Pradesh, India which examined violence from husbands and clients found an association between husband-perpetrated violence and increased risk of inconsistent condom use with clients[25]. However, this study did not examine prevalence of biological outcomes (HIV/STI prevalence) and did not include non-marital intimate partners or other workplace/community perpetrators. FSWs also face violence in their wider community. Previously, violence from ‘rowdies’ (gang leaders/members) and ‘strangers’ has been reported in India[7 8] but there is currently no research on how violence in the community impacts on HIV/STI risk. Additionally no studies have examined the risks associated with experiencing violence from multiple perpetrators i.e. from domestic *and* non-domestic (workplace/community) perpetrators. As a result, there is a need to better understand how violence from different and/or multiple perpetrators impacts on HIV/STI infection and sexual risk behaviours among FSWs.

This study aims to address this gap in the current literature by describing the distribution of workplace, community and domestic perpetrators of violence among FSWs in Karnataka and examining whether HIV/STI infection and sexual risk behaviours differ depending on the perpetrator of violence.

For peer review only

METHODS

Study Design

Data were collected from two districts (Shimoga and Bangalore) in the third round of a series of IBBA surveys, in Karnataka state. Intervention programmes were first implemented in 2004. Round 3 IBBA surveys took place in July and August 2011[7].

Sample size calculations have been reported previously[8]. In brief, the target sample for each IBBA district was fixed at 400. To represent the greater number of FSWs in Bangalore and the variation in sex work typology, a sample size of 800 was used [8 26]. Following mapping of FSWs across the two districts, two different sampling methods were used. For FSWs working at brothels, lodges, homes, and *dhabas* (road-side eating establishments) with a more fixed population, a conventional cluster sampling method was used. For street-based FSWs, time-location cluster sampling was utilised. Inclusion criteria were women aged 18-49 years who had received money or gifts in exchange for sex at least once in the past month. FSWs gave written or witnessed verbal informed consent and were interviewed by trained female interviewers in a rented room close to their workplace[8 26 27]. No identifying information was recorded.

The behavioural questionnaire was initially prepared in English and then translated into the local language, Kannada. It included one question on non-partner physical violence [*"In the last six months, how many times would you say someone has beaten you? (hurt, hit, slapped, pushed, kicked, punched, choked, burned?) Who did this to you?"]* and one question on non-partner sexual violence *("in the past one year, has anyone besides your main partner ever forced you to have sexual intercourse when you did not want to? If yes, who was/were this/these person/s?")*[7]. Women were given a pre-defined list of perpetrators to select from as well as the option to qualitatively report 'other' perpetrators. In round 3 in Bangalore and Shimoga, detailed questions on physical (6 items) and sexual violence from non-paying intimate partners (2 items) in the last 12 months were also included based on WHO operational definitions of violence[28] (Appendix A). Due to the two different timeframes (6 and 12 months), the term 'recent' violence will refer to the past 6/12 months.

Laboratory Methods

Blood samples were taken to test for HIV and syphilis. A confirmed syphilis infection was defined by having a Rapid Plasma Reagin (RPR) positive and a Treponema

For peer review only - http://bmjopen.bmj.com/site/about/guidelines.xhtml

Pallidum Haemagglutination Assay (TPHA) positive test with an RPR titre of greater than 1:8 classified as high-titre syphilis; high-titre syphilis is indicative of recent syphilis infection. Further details of laboratory methods have been previously reported[27].

Statistical Analyses

The analysis was carried out in STATA 13.1. To take account of sampling probabilities at district, primary sampling unit, and individual levels, as well as rates of non-response, data were appropriately weighted. The main exposure, violence, was categorised into workplace perpetrators (clients, police, pimps, madams and co-workers); community perpetrators (strangers, rowdies, neighbours, auto drivers, assistant ward boys, friends and relatives); and domestic perpetrators (husbands, regular partners and lovers). This classification was based on assumptions about which environment (domestic, workplace or community) violence is most likely to have been perpetrated in. In our preliminary analysis we examined community and workplace violence separately but found the results were very similar; due to the small number of community perpetrators, we therefore decided to collapse this into one category, to create 4 categories of exposure – 'no violence', 'domestic violence only', 'workplace and/or community violence only' and 'domestic *and* workplace/community violence'. The primary outcomes were HIV, syphilis and STI symptom prevalence. Secondary outcomes included condom use at last sex; condom breakage at last sex; client or FSW under the influence of alcohol during last sex; STI clinic visit in the past 6 months; and contact with a peer educator in the past month. Associations were measured using odds ratios (ORs) and p-values were obtained using the Wald chi-square test. As the data was weighted and analysed using survey set commands, we used a joint hypothesis test, the adjusted Wald test to obtain p-values using testparm in Stata. This tests the null hypothesis that the co-efficients are simultaneously equal to zero, and therefore tests whether there is variation between categories of exposure to violence. For multivariate analysis, age and district were selected as *a priori* confounders. Confounders were identified separately for each outcome using a change-in-estimate approach, but to increase the uniformity of the multivariate models, all outcomes were finally adjusted for the same variables. We did not adjust each outcome for all the other outcomes due to co-linearity between many of the main outcomes. The adjusted Wald test was used to test for effect modification.

For peer review only

For peer review only - http://bmjopen.bmj.com/site/about/guidelines.xhtml

Ethical Considerations

This study was approved by the ethical review board of St Johns Medical College, Bangalore, India (IRB: 179/2010); the Research Ethics Board at the University of Manitoba, Canada (IRB: H2005:098); and the Research Ethics Committee at the London School of Hygiene and Tropical Medicine (IRB: 11118).

Patient and Public Involvement

FSW community based organisations (CBOs), Implementing Partners working with FSWs and peer educators who were sex workers were involved in the design of the IBBA questionnaire and recruitment of women. The IBBA results, including the violence analyses, were disseminated to the community via presentations to the CBOs and Implementing Partners.

For peer review only

RESULTS

Study Population and Violence Experience

Overall, 1111 FSWs participated in the study [Shimoga (n=393), Bangalore (n=718]. Over one-third (34.9%) of FSWs reported recent (past 6/12 months) physical and/or sexual violence with recent physical violence (29.6%) more prevalent than recent sexual violence (21.9%) (Table 1). Reported domestic violence experience was high, with sixty percent of FSWs reporting intimate partner violence (IPV) in their lifetime and over a quarter of women (27.1%) reporting recent domestic violence (past 12 months). Recent workplace violence (past 6 months) was reported by 11.1% of FSWs, with sexual violence (8.2%) more prevalent than physical violence (5.4%). Workplace violence was mainly perpetrated by clients (9.2%), with <1% perpetrated by police, co-workers, and pimps. Recent violence by perpetrators from the community (past 6 months) was the least prevalent (4.1%) and was perpetrated mainly by strangers (2.1%) and 'rowdies' (1.1%) (Table 1).

The venn diagram in Fig. 1 shows the proportion of women experiencing violence from different perpetrators, and the overlap between violence experienced by workplace, community and domestic perpetrators. Thus, of the 34.9% of FSWs who reported recent violence, 6.8% reported violence by 2 or more different perpetrator types, and 6.2% reported violence by domestic *and* workplace or community perpetrators.

Table 1 Physical and sexual violence by perpetrator

Type of violence, by perpetrator		Recent Physical Violence %	Recent Sexual Violence[1] %	Recent physical and/or sexual violence %
Overall		29.6	21.9	34.9
Recent domestic violence		25.1	14.7	27.1
	Husband/regular partner	25.1	14.7	27.1
Recent workplace violence		5.4	8.2	11.1
	Client	4.0	7.2	9.2
	Police	0.5	0.9	0.9
	Co-worker	1.0	0.0	1.0
	Pimp	0.0	0.2	0.2
Recent community violence		2.7	2.9	4.2
	Strangers	1.6	1.1	2.1
	Rowdies*	0.7	1.0	1.1

Neighbours	0.3	0.0	0.3
Auto driver	0.1	0.0	0.1
Assistant ward boy**	0.0	0.05	0.1
Relatives	0.2	0.4	0.5
Friends	0.0	0.4	0.4

Missing observations: [1] n=19 (1.7%)
*Rowdies: a member or leader of a gang, who has committed offences punishable under the Indian Penal Code
**Assistant Ward Boy: healthcare worker

The mean age of respondents was 32.9 years, and 54.5% were illiterate (Table 2). Two-thirds (66.2%) had a regular partner, and the majority of women had at least one child. Two-thirds (66.1%) had an additional income to sex work. Women solicited clients either by phone (56.7%) or from public places (32.5%). The median number of clients entertained per week was 6 [range 1-70; interquartile range (IQR) 4, 10] and 15.6% had ever practiced sex work outside the district.

Due to the small number of women who reported community violence, for the remaining analyses, workplace and community violence were combined into one category ‘workplace / community violence’. Amongst FSWs who experienced recent violence, socio-demographic and sex work characteristics differed by the perpetrator of violence (Table 2). Women who reported recent workplace/community violence were more likely to solicit clients from public places (53.7%), whereas women who reported recent domestic violence only were more likely to solicit clients by phone (53.9%). A higher median number of clients per week was reported among women who experienced workplace/community violence (9; IQR: 5-12) or violence by both domestic *and* workplace/community perpetrators (9; IQR: 6-15), and these women were more likely to have migrated for sex work compared with women who had experienced domestic violence only or no violence (Table 2). Women who reported recent violence by both domestic *and* workplace/community perpetrators had the lowest mean age at start of sex work (25.4 years) and lowest mean age at first sex (15.4 years).

Table 2 Socio-demographic and sex work characteristics of FSWs in Shimoga and Bangalore and associations with violence by perpetrator

Characteristic	Overall	Recent violence by perpetrator

For peer review only - http://bmjopen.bmj.com/site/about/guidelines.xhtml

			**No violence**	**Domestic violence only**	**Workplace and/or community violence only**	**Domestic and workplace/ community violence**	**P value (chi square test)**
			%(n=727)	*%(n=216)*	*%(n=80)*	*%(n=69)*	
Age, years	<25	13.1	12.2	13.3	22.0	12.7	0.18
	25-29	22.4	20.9	24.6	21.9	36.1	
	30-39	45.2	44.6	47.4	41.0	42.7	
	40+	19.3	22.4	14.6	15.1	8.5	
	Mean	32.9	33.4	32.2	31.1	30.8	
Literacy	Illiterate	54.5	56.2	56.2	37.8	57.9	0.07
Marital Status	Lives alone	44.2	53.3	8.3	78.7	18.1	<0.0001
	Lives with partner other than husband	4.5	4.4	4.6	5.8	4.6	
	Married and lives with husband	51.2	42.3	87.0	15.5	76.2	
Regular partner	Yes	66.2	58.3	95.1	46.6	88.2	<0.0001
Number of children	0	9.7	8.7	7.3	22.5	12.2	0.03
	1-2	60.3	62.0	60.1	57.8	53.9	
	3+	30.1	29.4	32.6	19.7	34.0	
	Mean	2.0	2.0	2.2	1.7	2.0	
District	Bangalore	50.8	54.0	42.1	64.1	50.2	0.022
	Shimoga	49.3	46.0	57.9	35.9	49.8	
Additional income to sex work[1]	Yes	66.1	67.5	67.7	58.5	59.5	0.31
Age at first sex (years)	<15	48.2	49.4	44.9	38.9	64.6	0.07
	15+	51.8	50.6	55.1	61.1	35.4	
	Mean	16.1	16.1	16.3	16.6	15.4	
Age started sex work (years)	<20	5.5	5.6	4.8	8.2	5.7	0.056
	20-24	25.2	22.5	26.8	31.1	37.6	
	25-29	29.0	26.8	33.5	30.9	32.5	
	30+	40.3	45.1	35.0	29.8	24.3	
	Mean	28.3	28.9	27.7	26.9	25.4	
Place of solicitation of sex work	Home	7.4	8.4	7.9	3.1	4.7	0.0008
	Rented room/lodge/brothel	3.4	2.6	6.7	0.6	2.4	
	Public place/tamasha/other	32.5	28.3	31.5	53.7	43.4	
	Phone	56.7	60.7	53.9	42.6	49.4	
How much charged for sex with last client	400+	53.1	53.1	52.2	62.6	46.7	0.43
	Mean	459.3	469.8	442.5	458.5	422.3	

For peer review only

(rupees)							
Number of clients/ week	1-4	28.4	28.2	34.4	18.2	12.8	<0.0001
	5-9	45.0	46.6	47.3	37.7	42.0	
	10+	26.6	25.2	18.3	44.1	45.2	
	Median	6.0	6.0	6.0	9.0	9.0	
Migrant sex work (ever practiced sex work outside the district and/or in Mumbai)	Yes	15.6	12.6	11.9	39.9	25.8	<0.0001

Missing observations: [1] n=6(0.5%), [2] n=1(0.1%)

HIV/STI risk

Overall HIV prevalence was 8.2%, reactive syphilis 3.1% and high-titre syphilis 0.5%. In multivariate analysis, there was no evidence of an association between violence by perpetrator and either HIV (P-value: 0.27) or reactive syphilis (P-value: 0.76) (Table 3). However, there was strong evidence (P-value <0.0001) for an increased odds of high-titre syphilis infection amongst women who reported recent violence by both domestic *and* workplace/community perpetrators compared with women who reported no recent violence (aOR: 24.96; 95% CI: 5.94-96.70).

Self-report of STI symptoms (vaginal discharge/genital ulcers/abdominal pain not associated with menses) in the past year was higher amongst women who reported recent violence compared to FSWs who reported no violence. In multivariate analyses, there was strong evidence for an increased odds of STI symptoms in all categories of violence by perpetrator, with those who experienced violence by both domestic *and* workplace/community perpetrators having the highest odds of STI symptoms (aOR: 3.90; 95% CI: 2.10-7.26) (Table 3).

Condom Use

Recent violence by a specific perpetrator was associated with reduced condom use in that setting (Table 3). In adjusted analyses, any recent violence experience, regardless of the perpetrator, was associated with a significant reduction in reported condom use at last sex with occasional and regular clients. In multivariate analysis, recent violence experience by workplace perpetrators, or by domestic *and* workplace/community perpetrators, was significantly associated with reduced condom use with last occasional client and last regular client, compared with women reporting no recent violence. Overall, just one-fifth (19.5%) of FSWs reported condom use at last sex with a regular partner. Reported condom use with regular

For peer review only

partners was lower amongst women reporting recent domestic violence compared with women reporting no recent domestic violence, although this association did not remain significant in multivariate analyses.

Condom breakage at last sex was more likely among women who reported any recent violence (5.1%) compared to those who did not report recent violence (1.2%). In multivariate analysis there was strong evidence (P-value: 0.0001) for increased condom breakage among women who reported recent domestic violence (aOR: 3.72; 95%CI: 1.13-12.25), with the highest odds amongst women who reported violence by both domestic *and* workplace/community perpetrators (aOR: 19.29; 95%CI: 5.42-68.73).

Alcohol use

In univariate and adjusted analyses women who reported recent violence by workplace/community perpetrators (53.9%; aOR: 1.66; 95%CI: 0.96-2.84; P value: 0.024) and by both domestic *and* workplace/community perpetrators (56.0%; aOR: 2.16; 95%CI: 1.19-3.92, P-value: 0.024) were more likely to report either themselves, their client or both being under the influence of alcohol at last sex compared to women who reported no violence or domestic violence only.

Programme Exposure

Women who reported any recent violence were more likely to have visited an STI clinic in the last six months (44.5%) compared to those who did not report recent violence (27.8%) with the highest aOR amongst those who reported recent violence by both domestic *and* workplace/community perpetrators (aOR: 3.18; 95% CI: 1.68-6.03). Women who had experienced any recent violence (96.9%) were more likely to have had contact with a peer educator in the past month compared to women who had not experienced recent violence (92.0%), with some evidence for this association in multivariate analyses (aOR: 2.22; 95% CI: 0.98-5.00; P-value: 0.055).

For peer review only - http://bmjopen.bmj.com/site/about/guidelines.xhtml

Table 3 Violence by perpetrator and associations with HIV/STI prevalence and sexual risk behaviours

		Recent violence from any perpetrator			Violence by perpetrator (reference group: no recent violence)			
		**No recent violence** *%(n=727)*	**Any recent violence** *%(n=365)*	**P value**	**Domestic violence only** *%(n=216)*	**Workplace and/or community violence only** *%(n=80)*	**Domestic *and* workplace or community violence** *%(n=69)*	**P value***
HIV	%	8.1	6.1		2.5	13.4	8.9	
	Crude OR	1.0	0.73(0.42-1.27)	0.26	0.28(0.11-0.73)	1.75(0.88-3.5.0)	1.11(0.40-3.38)	0.022
	Adjusted OR	1.0	0.82(0.44-1.53)	0.53	0.40(0.15-1.09)	1.16(0.55-2.44)	1.32(0.41-4.29)	0.27
Reactive syphilis[1]	%	3.4	2.9		1.5	6.2	3.5	
	Crude OR	1.0	0.87(0.40-1.91)	0.74	0.42(0.09-2.02)	2.04(0.60-6.89)	1.12(0.30-4.22)	0.60
	Adjusted OR	1.0	1.27(0.68-2.38)	0.46	1.14(0.30-4.46)	1.17(0.57-2.40)	2.04(0.53-7.81)	0.76
High titre syphilis (recent syphilis)	%	0.38	0.64		0	0.9	2.5	
	Crude OR	1.0	1.70(0.36-8.03)	0.50	-	2.36(0.25-22.07)	6.74(1.15-39.58)	0.11
	Adjusted OR	1.0	2.22(0.54-9.17)	0.27	-	2.27(0.26-19.8)	24.96(5.94-96.70)	<0.0001
STI symptoms in past 12 months (vaginal discharge, lower abdominal pain not associated with menses and/or genital ulcer)	%	30.7	48.9		41.5	57.3	63.3	
	Crude OR	1.0	2.16(1.61-2.89)	<0.0001	1.60(1.11-2.31)	3.03(1.77-5.18)	3.90(2.18-6.95)	<0.0001
	Adjusted OR	1.0	2.27(1.66-3.09)	<0.0001	1.87(1.24-2.81)	2.41(1.40-4.17)	3.90(2.10-7.26)	<0.0001
Condom use last sex with occasional client[2]	%	97.5	94.5		97.2	91.6	91.0	
	Crude OR	1.0	0.45(0.21-0.96)	0.038	0.87(0.29-2.63)	0.28(0.10-0.74)	0.26(0.09-0.75)	0.0073
	Adjusted OR	1.0	0.39(0.19-0.83)	0.014	1.03(0.33-3.28)	0.20(0.07-0.52)	0.22(0.06-0.81)	0.0001

For peer review only - http://bmjopen.bmj.com/site/about/guidelines.xhtml

Condom use last sex with regular client[3]	%	93.0	88.2		92.6	85.9	76.0	
	Crude OR	1.0	0.56(0.32-0.98)	0.043	0.94(0.44-2.01)	0.46(0.20-1.05)	0.24(0.11-0.50)	0.0012
	Adjusted OR	1.0	0.61(0.32-1.15)	0.12	1.25(0.54-2.90)	0.33(0.15-0.73)	0.25(0.10-0.59)	0.0003
Condom use at last sex with regular partner	%	23.1	13.8		12.2	27.8	10.4	
	Crude OR	1.0	0.53(0.32-0.89)	0.016	0.46(0.26-0.84)	1.29(0.51-3.22)	0.39(0.16-0.93)	0.012
	Adjusted OR	1.0	0.63(0.35-1.14)	0.13	0.79(0.42-1.51)	0.40(0.14-1.12)	0.48(0.14-1.67)	0.30
Condom breakage at last sex[4]	%	1.2	5.1		3.0	3.1	15.1	
	Crude OR	1.0	4.38(1.91-10.02)	0.0005	2.46(0.84-7.25)	2.60(0.62-10.9)	14.3(5.10-40.30)	<0.0001
	Adjusted OR	1.0	4.32(1.74-10.73)	0.0017	3.72(1.13-12.25)	1.71(0.36-8.20)	19.29(5.42-68.73)	0.0001
Either client, FSW or both under the influence of alcohol at last sex	%	35.8	42.4		34.1	53.9	56.0	
	Crude OR	1.0	1.32(0.99-1.76)	0.058	0.93(0.661.30)	2.09(1.24-3.52)	2.28(1.31-3.99)	0.0015
	Adjusted OR	1.0	1.29(0.09-1.77)	0.12	0.97(0.66-1.42)	1.66(0.96-2.84)	2.16(1.19-3.92)	0.024
Visited an STI clinic in past six months for STI symptoms[5]	%	27.8	44.5		39.0	51.6	53.2	
	Crude OR	1.0	2.08(1.49-2.92)	<0.0001	1.66(1.10-2.52)	2.77(1.61-4.78)	2.95(1.61-5.43)	<0.0001
	Adjusted OR	1.0	2.28(1.59-3.27)	<0.0001	2.04(1.28-3.24)	2.32(1.35-3.97)	3.18(1.68-6.03)	0.0001
Had contact with a peer educator in the last month[6]	%	92.0	96.9		96.3	100	95.2	
	Crude OR	1.0	2.74(1.24-6.07)	0.013	2.27(0.86-6.00)	-	1.73(0.55-5.43)	0.20
	Adjusted OR	1.0	2.22(0.98-5.00)	0.055	1.75(0.63-4.90)	-	1.18(0.36-3.92)	0.56

Models adjusted for age, district, marital status, migrant sex work, place of selling sex and having an income other than sex work;
Missing observations: [1] n=1 (0.1%), [2] n=1(0.1%), [3] n=1(0.1%), [4] n=3(0.3%), [5] n=159 (14.3%), [6] n=22(2.0%); *Adjusted Wald test: tests the null hypothesis that the co-efficients (categories of exposure to violence) are equal to zero

For peer review only - http://bmjopen.bmj.com/site/about/guidelines.xhtml

DISCUSSION

This is the first study globally, to our knowledge, to examine violence experience among FSWs by perpetrators in the workplace, community and home, and associations with biological outcomes and HIV/STI risk behaviours. We found a high prevalence of violence from a range of perpetrators experienced by FSWs in this setting in India, with recent domestic violence more commonly reported than violence by workplace or community perpetrators. Additionally, we found that HIV/STI risk differed by perpetrator of violence and was highest amongst women who reported recent violence from multiple perpetrators; women reporting violence by domestic *and* workplace/community perpetrators were significantly more likely to have high-titre syphilis infection and had the highest odds of recent STI symptoms, condom breakage at last sex, alcohol use at last sex and no condom use at last sex with regular clients. This study is the first of its kind to show that increased STI prevalence and HIV/STI risk among FSWs is associated with experience of violence from multiple perpetrators. It also adds to a growing body of research globally, reporting the burden and range of perpetrators of violence among FSWs.

The pathways between violence exposure and increased HIV/STI risk are complex. Theories of risk pathways include HIV/STI risk associated with forced sex with an HIV-infected partner, women fearing violence if they request condom use, and increased high-risk behaviours as a result of the psychological impact of sexual/physical abuse[2 29]. In South Africa, HIV/STI risks among women from the general population have been found to be highest amongst those experiencing the highest levels of violence[30 31], suggesting a 'dose-response' effect between violence and HIV/STI risk. Although our study did not measure levels of violence, it is possible that women who experience violence from multiple perpetrators experience more violence overall than those who experience violence from domestic or workplace perpetrators only. This may partly explain the increased STI prevalence and sexual risk behaviours amongst women in our study who reported violence from multiple perpetrators.

Despite the high rates of domestic violence, our study findings suggest that violence by workplace/community perpetrators is more important for increasing sexual risk behaviours overall and during sex work, compared with domestic violence. Although a previous study with FSWs in India reported an association between husband-perpetrated violence and reduced condom use with clients[25], in our study, we found no associations between domestic violence and sexual risk behaviours in the

For peer review only - http://bmjopen.bmj.com/site/about/guidelines.xhtml

workplace, such as condom use with clients. However, women in our study who reported domestic violence only did have increased odds of STI symptoms and condom breakage at last sex compared to women who did not report any recent violence, suggesting that domestic violence is associated with some level of increased HIV/STI risk.

A recent systematic review of domestic violence among women in India estimated the median prevalence of lifetime and domestic violence in the past year was 41% and 30%, respectively[32]. In our study FSWs reported a much higher prevalence of lifetime (60.1%) violence and similar rates of recent domestic violence (27.1%). These high levels of domestic violence need to be addressed to reduce impacts on physical and psychological health[33]. So far, HIV prevention programmes with FSWs have focused mainly on reducing workplace violence[7 8 21] and improving condom use with clients[26]. Although there are examples of successful interventions to reduce domestic violence in women in the general population[34], the efficacy of such interventions among FSWs is unknown. A cluster RCT with FSWs, in Karnataka India, aimed at reducing IPV and improving condom use with their lover/husband is currently being assessed, and is the first of its kind to address domestic violence among FSWs[35].

Prevalence of recent workplace violence was relatively high (11.1%) despite the success of recent violence interventions in Karnataka[7], with clients the major perpetrators. Reported community violence was low (4.1%) compared to violence from other perpetrators. However, FSWs may be at greater risk of violence from community perpetrators compared to women in the general population, due to stigma and dangerous working environments. An important finding was the strong association between having experienced violence by both domestic *and* workplace/community perpetrators and increased odds of high-titre syphilis, demonstrating biological evidence of increased STI risk. As high-titre syphilis infection indicates recent infection, the direction of the association is more plausible compared to measures of chronic STI infection (HIV and reactive syphilis). Unfortunately in IBBA R3, FSWs were not tested for other incident STIs, due to budget constraints, although violence has been associated with gonorrhea in previous IBBAs[7]. Self-reported STI symptoms were strongly associated with violence from all perpetrators with the highest odds amongst those who reported both domestic *and* workplace/community violence. Although this may indicate STI infection in some cases, self-reported STI symptoms are not a reliable indicator of

For peer review only

biological infection[36]. Vaginal discharge in women in India has been linked to depression and psychosocial stress, which may partly explain this association[37]. The reduced odds of condom use with clients amongst women who reported violence by workplace/community perpetrators and by both domestic *and* workplace/community perpetrators, but not domestic violence, indicates that the association between violence and HIV/STI risk may be driven by the environment in which the violence occurs (although this is based on the assumption that different perpetrators are associated with particular environments).

The finding that FSWs who report recent violence have higher STI clinic attendance and recent contact with a peer educator reflects positively on the HIV/STI prevention programme in Karnataka, suggesting recent experience of violence does not hinder women from accessing services. In this study, having experienced workplace/community violence and both domestic *and* workplace/community violence was associated with alcohol use at last sex. Having experienced violence can lead to increased alcohol consumption as a coping mechanism[19 21]. Alternatively being under the influence of alcohol may increase vulnerability to violence and arrest[19].

This study had strengths and limitations. Although previous research has examined IPV and workplace violence among FSWs[25], none have included community violence or examined associations with biological STI infection. Only one previous study in Soweto, South Africa has reported on the prevalence of violence experience from multiple perpetrators among FSWs[10], but this study did not examine associations with HIV/STI prevalence or risk behaviours. To our knowledge, our study is the first to demonstrate increased prevalence of STI infection and sexual risk behaviours among FSWs who experience violence from multiple perpetrators, compared with FSWs who report either no recent violence, or recent violence from domestic or workplace perpetrators only. Although the data were collected in 2011, they remain the most recent data on HIV/STI prevalence and risk behaviours among FSWs in Karnataka. In addition, this is one of the few datasets available globally, which assesses exposure to both workplace and domestic violence among FSWs, as well as biological and behavioural markers of HIV and STI risk and infection. Other important strengths were the robust sampling strategy and the reasonably large sample size. With cross-sectional data, it is not possible to ascertain the direction of association for some outcomes or infer causality. Reporting bias may have contributed to over-reporting of certain outcomes (such as condom use) while more

For peer review only - http://bmjopen.bmj.com/site/about/guidelines.xhtml

stigmatized and sensitive topics (such as alcohol consumption and violence) may have been under-reported. The categorisation of violence by perpetrators was based on crude definitions, which likely do not reflect the fluidity of relationships and environments in which the violence occurs. For example, women who sell sex at home may experience domestic and workplace violence in one physical environment while the definition between regular client and lover/partner can become blurred, with clients becoming lovers and vice versa. Some associations may have been due to chance, particularly for outcomes with small numbers and wide confidence intervals, such as high titre syphilis infection. Additionally overlaps in confidence intervals between the exposure categories, indicate there is uncertainty in whether there is a true difference in risk by perpetrator of violence. If the WHO standardized 13-item violence questionnaire, which has been shown to yield higher response rates[8], had also been used for non-partners, it might have increased reporting of violence from workplace and community perpetrators. There was a discrepancy in the timeframe between recent non-partner physical violence (past 6 months) and recent non-partner sexual violence/intimate partner violence (past 12 months), which could have led to under-reporting of non-partner physical violence.

Despite these limitations, the findings of this study have important implications for HIV/STI prevention among FSWs. Violence against FSWs across both domestic, workplace and community settings needs to be addressed through integrated, comprehensive HIV programmes to enforce their human right to be able to live and work without fear for their safety.

For peer review only

Contributors:

AB conducted the analyses, and wrote the first draft of the manuscript. RP supervised the analyses and reviewed the article. RP, SI, HLM, LP, JB, and SM contributed to the study design and reviewed the article. TSB conceptualised the study, supervised the analyses, and reviewed the article. The authors thank the women who participated in this study.

Funding:

This study was supported by the India AIDS Initiative (*Avahan*) of the Bill & Melinda Gates Foundation, grant no. OPP52138. Tara Beattie, Ravi Prakash, Lucy Platt and Shajy Isac were supported by the UK Department for International Development (DFID) as part of STRIVE, a 6-year programme of research and action devoted to tackling the structural drivers of HIV (http://STRIVE.lshtm.ac.uk/). Tara Beattie is supported by a British Academy Fellowship. The views expressed herein are those of the authors and do not necessarily reflect the official policy or position of the Bill and Melinda Gates Foundation, UK DFID or the British Academy.

Competing Interests:

None

Data Sharing Statement:

No additional data are available.

For peer review only

REFERENCES

1. Gupta GR, Parkhurst JO, Ogden JA, et al. Structural approaches to HIV prevention. *Lancet* 2008;372(9640):764-75. doi: 10.1016/s0140-6736(08)60887-9 [published Online First: 2008/08/09]
2. Dunkle KL, Decker MR. Gender-based violence and HIV: reviewing the evidence for links and causal pathways in the general population and high-risk groups. *Am J Reprod Immunol* 2013;69 Suppl 1:20-6. doi: 10.1111/aji.12039
3. Deering KN, Amin A, Shoveller J, et al. A systematic review of the correlates of violence against sex workers. *Am J Public Health* 2014;104(5):e42-54. doi: 10.2105/ajph.2014.301909 [published Online First: 2014/03/15]
4. Devries KM, Mak JYT, García-Moreno C, et al. The Global Prevalence of Intimate Partner Violence Against Women. *Science* 2013;340(6140):1527-28. doi: 10.1126/science.1240937
5. Baral S, Beyrer C, Muessig K, et al. Burden of HIV among female sex workers in low-income and middle-income countries: a systematic review and meta-analysis. *The Lancet Infectious Diseases* 2012;12(7):538-49.
6. Decker MR, Crago A-L, Chu SKH, et al. Human rights violations against sex workers: burden and effect on HIV. *The Lancet*;385(9963):186-99. doi: 10.1016/S0140-6736(14)60800-X
7. Beattie TS, Bhattacharjee P, Isac S, et al. Declines in violence and police arrest among female sex workers in Karnataka state, south India, following a comprehensive HIV prevention programme. *J Int AIDS Soc* 2015;18:20079. doi: 10.7448/IAS.18.1.20079
8. Beattie TS, Bhattacharjee P, Ramesh BM, et al. Violence against female sex workers in Karnataka state, south India: impact on health, and reductions in violence following an intervention program. *BMC Public Health* 2010;10:476. doi: 10.1186/1471-2458-10-476
9. Schwitters A, Swaminathan M, Serwadda D, et al. Prevalence of rape and client-initiated gender-based violence among female sex workers: Kampala, Uganda, 2012. *AIDS and behavior* 2015;19 Suppl 1:S68-76. doi: 10.1007/s10461-014-0957-y [published Online First: 2014/11/30]
10. Coetzee J, Gray GE, Jewkes R. Prevalence and patterns of victimization and polyvictimization among female sex workers in Soweto, a South African township: a cross-sectional, respondent-driven sampling study. *Glob Health Action* 2017;10(1):1403815. doi: 10.1080/16549716.2017.1403815 [published Online First: 2017/12/07]
11. Pando MA, Coloccini RS, Reynaga E, et al. Violence as a barrier for HIV prevention among female sex workers in Argentina. *PLoS ONE [Electronic Resource]* 2013;8(1):e54147.
12. Decker MR, Pearson E, Illangasekare SL, et al. Violence against women in sex work and HIV risk implications differ qualitatively by perpetrator. *BMC Public Health* 2013;13:876.
13. Decker MR, McCauley HL, Phuengsamran D, et al. Violence victimisation, sexual risk and sexually transmitted infection symptoms among female sex workers in Thailand. *Sexually Transmitted Infections* 2010;86(3):236-40.
14. Tounkara FK, Diabate S, Guedou FA, et al. Violence, condom breakage, and HIV infection among female sex workers in Benin, West Africa. *Sexually Transmitted Diseases* 2014;41(5):312-8.
15. Bradley J, Rajaram S, Moses S, et al. Female sex worker client behaviors lead to condom breakage: a prospective telephone-based survey in Bangalore, South India. *AIDS & Behavior* 2013;17(2):559-67.

For peer review only - http://bmjopen.bmj.com/site/about/guidelines.xhtml

16. Choi SY, Chen KL, Jiang ZQ. Client-perpetuated violence and condom failure among female sex workers in southwestern China. *Sexually Transmitted Diseases* 2008;35(2):141-6.
17. Shannon K, Strathdee SA, Shoveller J, et al. Structural and environmental barriers to condom use negotiation with clients among female sex workers: implications for HIV-prevention strategies and policy. *Am J Public Health* 2009;99(4):659-65. doi: 10.2105/ajph.2007.129858 [published Online First: 2009/02/07]
18. Li Q, Li X, Stanton B. Alcohol Use Among Female Sex Workers and Male Clients: An Integrative Review of Global Literature. *Alcohol and Alcoholism (Oxford, Oxfordshire)* 2010;45(2):188-99. doi: 10.1093/alcalc/agp095
19. Mbonye M, Rutakumwa R, Weiss H, et al. Alcohol consumption and high risk sexual behaviour among female sex workers in Uganda. *African journal of AIDS research : AJAR* 2014;13(2):145-51. doi: 10.2989/16085906.2014.927779 [published Online First: 2014/09/02]
20. Chersich MF, Bosire W, King'ola N, et al. Effects of hazardous and harmful alcohol use on HIV incidence and sexual behaviour: a cohort study of Kenyan female sex workers. *Global Health* 2014;10:22.
21. Biradavolu MR, Burris S, George A, et al. Can sex workers regulate police? Learning from an HIV prevention project for sex workers in southern India. *Social Science & Medicine* 2009;68(8):1541-47. doi: http://dx.doi.org/10.1016/j.socscimed.2009.01.040
22. Gurnani V, Beattie TS, Bhattacharjee P, et al. An integrated structural intervention to reduce vulnerability to HIV and sexually transmitted infections among female sex workers in Karnataka state, south India. *BMC Public Health* 2011;11:755. doi: 10.1186/1471-2458-11-755
23. Panchanadeswaran S, Johnson SC, Sivaram S, et al. Intimate partner violence is as important as client violence in increasing street-based female sex workers' vulnerability to HIV in India. *International Journal of Drug Policy*;19(2):106-12.
24. Deering KN, Bhattacharjee P, Bradley J, et al. Condom use within non-commercial partnerships of female sex workers in southern India. *BMC Public Health*;11 Suppl 6:S11.
25. Reed E, Erausquin JT, Groves AK, et al. Client-perpetrated and husband-perpetrated violence among female sex workers in Andhra Pradesh, India: HIV/STI risk across personal and work contexts. *Sexually Transmitted Infections* 2016 doi: 10.1136/sextrans-2015-052162
26. Ramesh BM, Beattie TS, Shajy I, et al. Changes in risk behaviours and prevalence of sexually transmitted infections following HIV preventive interventions among female sex workers in five districts in Karnataka state, south India. *Sexually Transmitted Infections* 2010;86 Suppl 1:i17-24.
27. Reza-Paul S, Beattie T, Syed HU, et al. Declines in risk behaviour and sexually transmitted infection prevalence following a community-led HIV preventive intervention among female sex workers in Mysore, India. *AIDS (London, England)* 2008;22 Suppl 5:S91-100. doi: 10.1097/01.aids.0000343767.08197.18 [published Online First: 2009/01/07]
28. World Health Organisation. WHO multi-country study on women's health and domestic violence against women: Initial results on prevalence, health outcomes and women's responses. Geneva, 2005.
29. Ulibarri MD, Strathdee SA, Lozada R, et al. Prevalence and correlates of client-perpetrated abuse among female sex workers in two Mexico-U.S. border cities. *Violence Against Women* 2014;20(4):427-45.
30. Jewkes RK, Dunkle K, Nduna M, et al. Intimate partner violence, relationship power inequity, and incidence of HIV infection in young women in South Africa: a cohort study. *Lancet* 2010;376(9734):41-8.

For peer review only

31. Dunkle KL, Jewkes RK, Brown HC, et al. Gender-based violence, relationship power, and risk of HIV infection in women attending antenatal clinics in South Africa. *Lancet* 2004;363(9419):1415-21. doi: 10.1016/s0140-6736(04)16098-4 [published Online First: 2004/05/04]
32. Kalokhe A, del Rio C, Dunkle K, et al. Domestic violence against women in India: A systematic review of a decade of quantitative studies. *Global Public Health* 2016:1-16. doi: 10.1080/17441692.2015.1119293
33. World Health Organisation, London School of Hygiene & Tropical Medicine, South African Medical Research Council. Global and regional estimates of violence against women: prevalence and health effects of intimate partner violence and non-partner sexual violence, 2013.
34. Pronyk PM, Hargreaves JR, Kim JC, et al. Effect of a structural intervention for the prevention of intimate-partner violence and HIV in rural South Africa: a cluster randomised trial. *Lancet* 2006;368(9551):1973-83. doi: 10.1016/s0140-6736(06)69744-4 [published Online First: 2006/12/05]
35. Beattie TS, Isac S, Bhattacharjee P, et al. Reducing violence and increasing condom use in the intimate partnerships of female sex workers: study protocol for Samvedana Plus, a cluster randomised controlled trial in Karnataka state, south India. *BMC Public Health* 2016;16:660. doi: 10.1186/s12889-016-3356-7
36. Vishwanath S, Talwar V, Prasad R, et al. Syndromic management of vaginal discharge among women in a reproductive health clinic in India. *Sexually Transmitted Infections* 2000;76(4):303-06. doi: 10.1136/sti.76.4.303
37. Patel V, Pednekar S, Weiss H, et al. Why do women complain of vaginal discharge? A population survey of infectious and pyschosocial risk factors in a South Asian community. *Int J Epidemiol* 2005;34(4):853-62. doi: 10.1093/ije/dyi072 [published Online First: 2005/04/19]

FIGURES

Figure 1 Proportional venn diagram showing overlapping of physical and/or sexual violence experiences among FSWs by perpetrator

APPENDICES

See supplementary files for Appendix A (violence questionnaire)

For peer review only

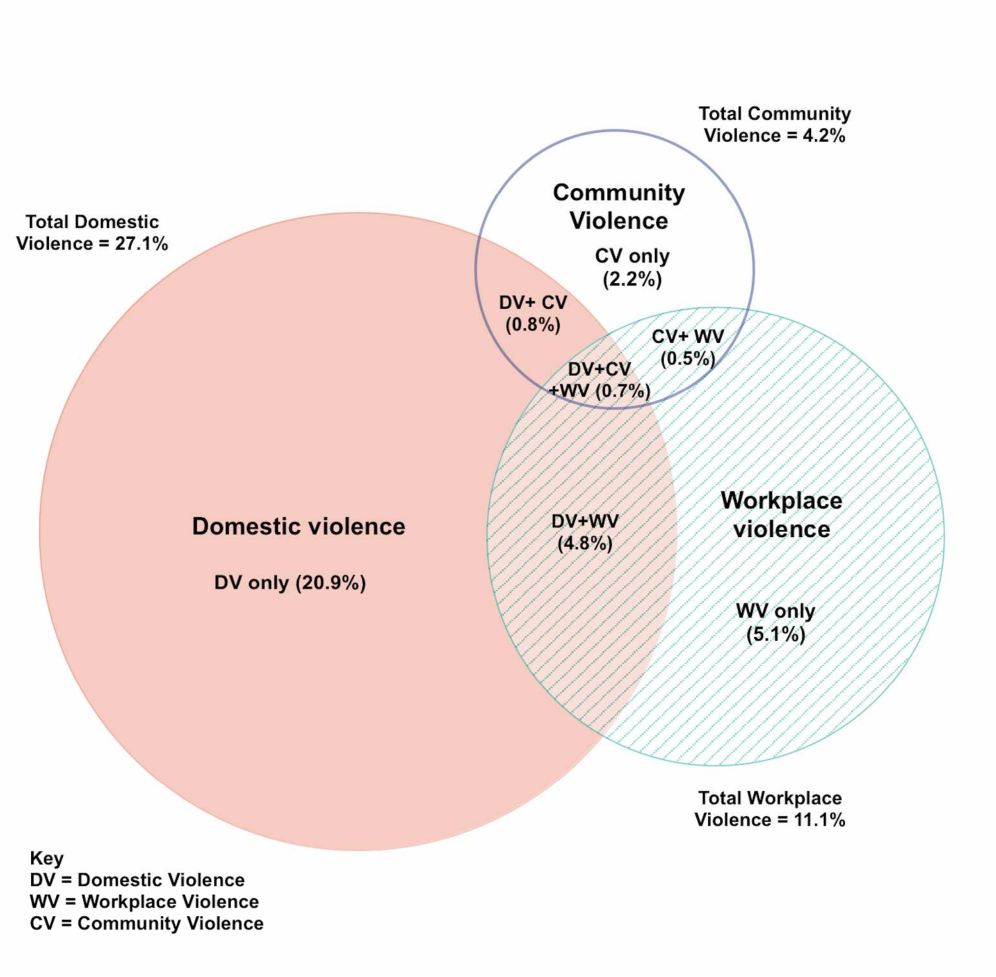

Figure 1 Proportional venn diagram showing overlapping of physical and/or sexual violence experiences among FSWs by perpetrator

260x255mm (300 x 300 DPI)

Appendix A: IBBA violence questionnaire

Physical and sexual violence from a husband/main partner based on WHO operational definitions of violence	Q717b: A. Has any husband or main partner that you have lived with ever done following things to you?<br><br>a) pushed you, shaken you, or thrown something at you?<br><br>b) slapped or shoved you?<br><br>c) hit you with his fist or something else that could hurt you?<br><br>d) kicked you, dragged you or beat you up?<br><br>e) tried to choke you or burn you on purpose<br><br>f) threatened to use or actually used a knife, gun or any other weapon?<br><br>g) physically forced to have sex with him even when you did not want.<br><br>h) used threats of violence or rejection to forced you to have sex with him when you did not want to?<br><br>B. How often has this happened during the last 12 months: often, only sometimes, or not at all?

For peer review only

STROBE checklist for manuscript: Violence experience by perpetrator and associations with HIV/STI risk and infection: a cross-sectional study among female sex workers in Karnataka, south India

STROBE Statement—Checklist of items that should be included in reports of ***cross-sectional studies***

	Item No	Recommendation	Reported on page #
**Title and abstract**	1	(*a*) Indicate the study's design with a commonly used term in the title or the abstract	2
		(*b*) Provide in the abstract an informative and balanced summary of what was done and what was found	2, 3
**Introduction**			
Background/rationale	2	Explain the scientific background and rationale for the investigation being reported	4, 5
Objectives	3	State specific objectives, including any prespecified hypotheses	4,5
**Methods**			
Study design	4	Present key elements of study design early in the paper	5, 6
Setting	5	Describe the setting, locations, and relevant dates, including periods of recruitment, exposure, follow-up, and data collection	6
Participants	6	(*a*) Give the eligibility criteria, and the sources and methods of selection of participants	6
Variables	7	Clearly define all outcomes, exposures, predictors, potential confounders, and effect modifiers. Give diagnostic criteria, if applicable	7
Data sources/ measurement	8*	For each variable of interest, give sources of data and details of methods of assessment (measurement). Describe comparability of assessment methods if there is more than one group	7
Bias	9	Describe any efforts to address potential sources of bias	7
Study size	10	Explain how the study size was arrived at	6
Quantitative variables	11	Explain how quantitative variables were handled in the analyses. If applicable, describe which groupings were chosen and why	7
Statistical methods	12	(*a*) Describe all statistical methods, including those used to control for confounding	7
		(*b*) Describe any methods used to examine subgroups and interactions	7
		(*c*) Explain how missing data were addressed	
		(*d*) If applicable, describe analytical methods taking account of sampling strategy	7
		(*e*) Describe any sensitivity analyses	
**Results**			
Participants	13*	(a) Report numbers of individuals at each stage of study—eg numbers potentially eligible, examined for eligibility, confirmed eligible, included in the study, completing follow-up, and analysed	8
		(b) Give reasons for non-participation at each stage	
		(c) Consider use of a flow diagram	
Descriptive data	14*	(a) Give characteristics of study participants (eg demographic, clinical, social) and information on exposures and potential confounders	9

For peer review only

		(b) Indicate number of participants with missing data for each variable of interest	9, 10, 14
Outcome data	15*	Report numbers of outcome events or summary measures	11, 12, 13, 14
Main results	16	(*a*) Give unadjusted estimates and, if applicable, confounder-adjusted estimates and their precision (eg, 95% confidence interval). Make clear which confounders were adjusted for and why they were included	11, 12, 13, 14
		(*b*) Report category boundaries when continuous variables were categorized	9, 10
		(*c*) If relevant, consider translating estimates of relative risk into absolute risk for a meaningful time period	
Other analyses	17	Report other analyses done—eg analyses of subgroups and interactions, and sensitivity analyses	
**Discussion**			
Key results	18	Summarise key results with reference to study objectives	15, 17
Limitations	19	Discuss limitations of the study, taking into account sources of potential bias or imprecision. Discuss both direction and magnitude of any potential bias	16. 17
Interpretation	20	Give a cautious overall interpretation of results considering objectives, limitations, multiplicity of analyses, results from similar studies, and other relevant evidence	15-17
Generalisability	21	Discuss the generalisability (external validity) of the study results	15, 17
**Other information**			
Funding	22	Give the source of funding and the role of the funders for the present study and, if applicable, for the original study on which the present article is based	18

*Give information separately for exposed and unexposed groups.

Note: An Explanation and Elaboration article discusses each checklist item and gives methodological background and published examples of transparent reporting. The STROBE checklist is best used in conjunction with this article (freely available on the Web sites of PLoS Medicine at http://www.plosmedicine.org/, Annals of Internal Medicine at http://www.annals.org/, and Epidemiology at http://www.epidem.com/). Information on the STROBE Initiative is available at www.strobe-statement.org.

For peer review only

BMJ Open

Violence experience by perpetrator and associations with HIV/STI risk and infection: a cross-sectional study among female sex workers in Karnataka, south India

Journal:	*BMJ Open*
Manuscript ID	bmjopen-2017-021389.R2
Article Type:	Research
Date Submitted by the Author:	04-Jul-2018
Complete List of Authors:	Beksinska, Alicja; London School of Hygiene and Tropical Medicine Department of Global Health and Development,<br>Prakash, Ravi; Karnataka Health Promotion Trust<br>Isac, Shajy; Karnataka Health Promotion Trust<br>Mohan, H L; Karnataka Health Promotion Trust<br>Platt, Lucy; London School of Hygiene and Tropical Medicine Faculty of Public Health and Policy, Department of Social and Environmental Health<br>Blanchard, James; University of Manitoba, Department of Community Health Sciences; University of Manitoba, Department of Medical Microbiology<br>Moses, Stephen; University of Manitoba, Community Health Sciences; University of Manitoba, Department of Medical Microbiology<br>Beattie, Tara; London School of Hygiene and Tropical Medicine Department of Global Health and Development
**Primary Subject Heading**:	Sexual health
Secondary Subject Heading:	HIV/AIDS, Epidemiology
Keywords:	HIV & AIDS < INFECTIOUS DISEASES, Female Sex Workers, Sexually transmitted infections, Violence

SCHOLARONE™
Manuscripts

VIOLENCE EXPERIENCE BY PERPETRATOR AND ASSOCIATIONS WITH HIV/STI RISK AND INFECTION: A CROSS-SECTIONAL STUDY AMONG FEMALE SEX WORKERS IN KARNATAKA, SOUTH INDIA

Alicja Beksinska[1], Ravi Prakash[2], Shajy Isac[2], H.L. Mohan[2], Lucy Platt[3], James Blanchard[4,5], Stephen Moses[4,5], Tara S Beattie[1]

Institution(s): [1]London School of Hygiene and Tropical Medicine, Department of Global Health and Development, London, United Kingdom, [2]Karnataka Health Promotion Trust, Bengaluru, India, [3]London School of Hygiene and Tropical Medicine, Department of Social and Environmental Health, Faculty of Public Health and Policy, London, United Kingdom, [4]University of Manitoba, Department of Community Health Sciences, Winnipeg, Canada, [5]University of Manitoba, Department of Medical Microbiology, Winnipeg, Canada

Corresponding author:
Alicja Beksinska
Department of Global Health and Development, London School of Hygiene and Tropical Medicine, 15-17 Tavistock Place, London WC1H 9SN, UK
Email address: A.Beksinska1@uni.bsms.ac.uk
Tel no.: 07796980683

Word count: 3997

For peer review only

ABSTRACT

Objectives

Female sex workers (FSWs) experience violence from a range of perpetrators, but little is known about how violence experience across multiple settings (workplace, community, domestic) impacts on HIV/STI risk. We examined whether HIV/STI risk differs by the perpetrator of violence.

Methods

An Integrated Biological and Behavioural Assessment (IBBA) survey was conducted among random samples of FSWs in two districts (Bangalore and Shimoga) in Karnataka state, south India, in 2011. Physical and sexual violence in the past 6 months, by workplace (client, police, co-worker, pimp) or community (stranger, rowdy, neighbour, auto-driver) perpetrators was assessed, as was physical and sexual intimate partner violence in the past 12 months. Weighted, bivariate and multivariate analyses were used to examine associations between violence by perpetrator and HIV/STI risk.

Results

1111 FSWs were included (Bangalore=718, Shimoga=393). Overall, 34.9% reported recent physical and/or sexual violence. Violence was experienced from domestic (27.1%), workplace (11.1%) and community (4.2%) perpetrators, with 6.2% of participants reporting recent violence from both domestic *and* non-domestic (workplace/community) perpetrators. Adjusted analysis suggests that experience of violence by workplace/community perpertrators is more important in increasing HIV/STI risk during sex work (lower condom use with clients; client or FSW under the influence of alcohol at last sex) than domestic violence. However, women who reported recent violence by domestic *and* workplace/community perpetrators had the highest odds of high-titre syphilis infection, recent STI symptoms and condom breakage at last sex, and the lowest odds of condom use at last sex with regular clients, compared with women who reported violence by domestic or workplace/community perpetrators only.

For peer review only - http://bmjopen.bmj.com/site/about/guidelines.xhtml

Conclusion

HIV/STI risk differs by the perpetrator of violence and is highest among FSWs experiencing violence in the workplace/community *and* at home. Effective HIV/STI prevention programmes with FSWs need to include violence interventions that address violence across both their personal and working lives.

Strengths and limitations

• This study is the first to examine the association between violence exposure from multiple perpetrators and HIV/STI risk and prevalence among FSWs.
• The study used a robust sampling strategy and had a reasonably large sample size (>1000 FSWs).
• Intimate partner violence (IPV) was assessed using the validated 8-item WHO IPV questionnaire. However, violence experience by other perpetrators was assessed used a 2-item question which may have led to under-reporting of workplace/community violence.
• The categorisation of violence by perpetrators was based on crude definitions, which likely do not reflect the fluidity of relationships (for example client to intimate partner and vice versa).
• Some associations may have been due to chance, particularly for outcomes with small numbers, such as syphilis infection.

For peer review only

INTRODUCTION

Violence, in particular, gender-based violence, is recognised as a risk factor for HIV and sexually transmitted infections (STIs)[1]. Female sex workers (FSWs) experience high levels of violence and HIV/STIs[2]. Recent estimates indicate FSWs have a lifetime violence prevalence of 41%-65%[3] compared to 27.8%-32.2%[4] amongst women in the general population as well as 13.5 (95%CI: 10.0-18.1) times the odds of HIV infection[5]. FSWs commonly experience violence on entry into sex work when they are at their most vulnerable[2]. FSWs can experience violence in their workplace from a range of perpetrators including police, clients, pimps and madams[6-10], as well as in their community from private militias, religious groups and others who may perceive sex workers to be ‘immoral’ and blame them for the spread of HIV and STIs[6]. FSWs also experience high levels of domestic violence, from intimate partners[11 12].

Violence against FSWs is associated with increased HIV/STIs[7 8] and STI symptoms[7 13], and can hinder HIV prevention programming[11]. Recent violence experience may directly increase HIV/STI risk through condom breakage/failure or condom non-use[14-16]. Furthermore, men who perpetrate violence against women are more likely to engage in high risk behaviours including having multiple sexual partners, high alcohol consumption and inconsistent condom use, and have an increased prevalence of HIV, STIs and STI symptoms. This puts their sexual partners at increased HIV/STI risk[2]. HIV vulnerability may be increased indirectly as fear of police violence or arrest may result in women not carrying condoms or working in more isolated, dangerous locations[17], and deter them from accessing sexual health services[6]. Alcohol use is common among FSW populations[18] and their clients and is associated with increased HIV/STI risk[19] and violence experience[20 21].

India has the third largest HIV epidemic globally, with prevalence rates among FSWs ranging from 2-38%. Karnataka state in south India has one of the highest HIV burdens among FSWs, with prevalence previously reaching >30% in some districts[7]. Although sex work per se is not illegal, many FSWs and police wrongly understood this to be the case and sex work is highly stigmatised[21 22]. Violence against FSWs has been identified as a key concern[8]. In 2003, the Karnataka Health Promotion Trust (KHPT), in partnership with the University of Manitoba, was established to scale up HIV prevention programming with ‘high-risk’ populations. At scale, the intervention worked with over 60,000 FSWs per annum using a rights-based approach to address violence, stigma, and poverty as part of comprehensive HIV

For peer review only

prevention programming[22]. Changes in behaviour and HIV and STI prevalence were assessed using serial Integrated Biological and Behavioural Assessment (IBBA) cross-sectional surveys.

Studies examining the association between violence and HIV/STI infection and sexual risk behaviours among FSWs have primarily focused on client violence. Although there is now evidence of how HIV prevention programmes among FSWs can effectively reduce violence from non-partners[7 8 21] there has been less research on the impacts of domestic violence on HIV/STI risk, or the efficacy of programmes targeting domestic violence among FSWs. The complexity of violence from different perpetrators and the associated HIV/STI risks are still unclear which hinders the ability of researchers and policy-makers to design violence prevention programmes. Qualitative research has suggested that domestic violence may be as important as workplace violence in contributing to HIV/STI risk[23] and FSWs report low levels of condom use with intimate partners[24]. To our knowledge only one previous study in Andhra Pradesh, India which examined violence from husbands and clients found an association between husband-perpetrated violence and increased risk of inconsistent condom use with clients[25]. However, this study did not examine prevalence of biological outcomes (HIV/STI prevalence) and did not include non-marital intimate partners or other workplace/community perpetrators. FSWs also face violence in their wider community. Previously, violence from ‘rowdies’ (gang leaders/members) and ‘strangers’ has been reported in India[7 8] but there is currently no research on how violence in the community impacts HIV/STI risk. Additionally no studies have examined the risks associated with experiencing violence from multiple perpetrators i.e. from domestic *and* non-domestic (workplace/community) perpetrators. As a result, there is a need to better understand how violence from different and/or multiple perpetrators impacts on HIV/STI infection and sexual risk behaviours among FSWs.

This study aims to address this gap in the current literature by describing the distribution of workplace, community and domestic perpetrators of violence among FSWs in Karnataka and examining whether HIV/STI infection and sexual risk behaviours differ depending on the perpetrator of violence.

For peer review only - http://bmjopen.bmj.com/site/about/guidelines.xhtml

METHODS

Study Design

Data were collected from two districts (Shimoga and Bangalore) in the third round of a series of IBBA surveys, in Karnataka. Intervention programmes were first implemented in 2004. Round 3 IBBA surveys took place in July and August 2011[7].

Sample size calculations have been reported previously[8]. In brief, the target sample for each IBBA district was fixed at 400. To represent the greater number of FSWs in Bangalore and the variation in sex work typology, a sample size of 800 was used [8 26]. Following mapping of FSWs across the two districts, two sampling methods were used. For FSWs working at brothels, lodges, homes, and *dhabas* (road-side eating establishments) with a more fixed population, a conventional cluster sampling method was used. For street-based FSWs, time-location cluster sampling was utilised. Inclusion criteria were women aged 18-49 years who had received money or gifts in exchange for sex at least once in the past month. FSWs gave written or witnessed verbal informed consent and were interviewed by trained female interviewers in a rented room close to their workplace[8 26 27]. No identifying information was recorded.

The behavioural questionnaire was prepared in English and then translated into the local language, Kannada. It included one question on non-partner physical violence [*"In the last six months, how many times would you say someone has beaten you? (hurt, hit, slapped, pushed, kicked, punched, choked, burned?) Who did this to you?"*] and one question on non-partner sexual violence *("in the past one year, has anyone besides your main partner ever forced you to have sexual intercourse when you did not want to? If yes, who was/were this/these person/s?")*[7]. Women were given a list of perpetrators to select from as well as the option to qualitatively report 'other' perpetrators. In round 3 in Bangalore and Shimoga, detailed questions on physical (6 items) and sexual violence from non-paying intimate partners (2 items) in the last 12 months were also included based on WHO operational definitions of violence[28] (Appendix A). Due to the two different timeframes (6 and 12 months), the term 'recent' violence will refer to the past 6/12 months.

Laboratory Methods

Blood samples were taken to test for HIV and syphilis. A confirmed syphilis infection was defined by having a Rapid Plasma Reagin (RPR) positive and a Treponema

For peer review only - http://bmjopen.bmj.com/site/about/guidelines.xhtml

Pallidum Haemagglutination Assay (TPHA) positive with an RPR titre of greater than 1:8 classified as high-titre syphilis; high-titre syphilis is indicative of recent syphilis infection. Further details of laboratory methods have been previously reported[27].

Statistical Analyses

The analysis was carried out in STATA 13.1. To take account of sampling probabilities at district, primary sampling unit, and individual levels, as well as rates of non-response, data were appropriately weighted. The main exposure, violence, was categorised into workplace perpetrators (clients, police, pimps, madams and co-workers); community perpetrators (strangers, rowdies, neighbours, auto drivers, assistant ward boys, friends and relatives); and domestic perpetrators (husbands, regular partners and lovers). This classification was based on assumptions about which environment (domestic, workplace or community) violence is most likely to have been perpetrated in. In our preliminary analysis we examined community and workplace violence separately but found the results were very similar; due to the small number of community perpetrators, we decided to collapse this into one category, to create 4 categories of exposure – 'no violence', 'domestic violence only', 'workplace and/or community violence only' and 'domestic *and* workplace/community violence'. The primary outcomes were HIV, syphilis and STI symptom prevalence (STI symptoms were self reported vaginal discharge, lower abdominal pain not associated with menses and/or genital ulcer in the past 12 months). Secondary outcomes included condom use at last sex; condom breakage at last sex; client or FSW under the influence of alcohol during last sex; STI clinic visit in the past 6 months; and contact with a peer educator in the past month. Associations were measured using odds ratios (ORs) and p-values were obtained using the Wald chi-square test. As the data was weighted and analysed using survey set commands, we used a joint hypothesis test, the adjusted Wald test to obtain p-values using testparm in Stata. This tests the null hypothesis that the co-efficients are simultaneously equal to zero, and therefore tests whether there is variation between categories of exposure to violence. For multivariate analysis, age and district were selected as *a priori* confounders. Confounders were identified separately for each outcome using a change-in-estimate approach, but to increase the uniformity of the multivariate models, all outcomes were finally adjusted for the same variables. We did not adjust each outcome for all the other outcomes due to co-linearity between many of the main outcomes. The adjusted Wald test was used to test for effect modification.

For peer review only - http://bmjopen.bmj.com/site/about/guidelines.xhtml

Ethical Considerations

This study was approved by the ethical review board of St Johns Medical College in Bangalore, India (IRB: 179/2010); the Research Ethics Board at the University of Manitoba, Canada (IRB: H2005:098); and the Research Ethics Committee at the London School of Hygiene and Tropical Medicine (IRB: 11118).

Patient and Public Involvement

FSW community based organisations (CBOs), implementing partners and FSW peer educators were involved in the design of the questionnaire and recruitment of women. The results were disseminated back to the community via presentations to the CBOs and the implementing partners.

For peer review only

RESULTS

Study Population and Violence Experience

Overall, 1111 FSWs participated in the study [Shimoga (n=393), Bangalore (n=718]. Over one-third (34.9%) of FSWs reported recent (past 6/12 months) physical and/or sexual violence with recent physical violence (29.6%) more prevalent than recent sexual violence (21.9%) (Table 1). Reported domestic violence experience was high, with sixty percent of FSWs reporting intimate partner violence (IPV) in their lifetime and over a quarter of women (27.1%) reporting recent domestic violence (past 12 months). Recent workplace violence (past 6 months) was reported by 11.1% of FSWs, with sexual violence (8.2%) more prevalent than physical violence (5.4%). Workplace violence was mainly perpetrated by clients (9.2%), with <1% perpetrated by police, co-workers, and pimps. Recent violence by perpetrators from the community (past 6 months) was the least prevalent (4.1%) and was perpetrated mainly by strangers (2.1%) and 'rowdies' (1.1%) (Table 1).

The venn diagram in Fig. 1 shows the proportion of women experiencing violence from different perpetrators, and the overlap between violence experienced by workplace, community and domestic perpetrators. Thus, of the 34.9% of FSWs who reported recent violence, 6.8% reported violence by 2 or more different perpetrator types, and 6.2% reported violence by domestic *and* workplace or community perpetrators.

Table 1 Physical and sexual violence by perpetrator

**Type of violence, by perpetrator**		**Recent Physical Violence %**	**Recent Sexual Violence[1] %**	**Recent physical and/or sexual violence %**
Overall		29.6	21.9	34.9
Recent domestic violence		25.1	14.7	27.1
	Husband/regular partner	25.1	14.7	27.1
Recent workplace violence		5.4	8.2	11.1
	Client	4.0	7.2	9.2
	Police	0.5	0.9	0.9
	Co-worker	1.0	0.0	1.0
	Pimp	0.0	0.2	0.2
Recent community violence		2.7	2.9	4.2
	Strangers	1.6	1.1	2.1
	Rowdies*	0.7	1.0	1.1

For peer review only - http://bmjopen.bmj.com/site/about/guidelines.xhtml

Neighbours	0.3	0.0	0.3
Auto driver	0.1	0.0	0.1
Assistant ward boy**	0.0	0.05	0.1
Relatives	0.2	0.4	0.5
Friends	0.0	0.4	0.4

Missing observations: [1] n=19 (1.7%)
*Rowdies: a member or leader of a gang, who has committed offences punishable under the Indian Penal Code
**Assistant Ward Boy: healthcare worker

The mean age of respondents was 32.9 years, and 54.5% were illiterate (Table 2). Two-thirds (66.2%) had a regular partner, and the majority of women had at least one child. Two-thirds (66.1%) had an additional income to sex work. Women solicited clients either by phone (56.7%) or from public places (32.5%). The median number of clients entertained per week was 6 [range 1-70; interquartile range (IQR) 4, 10] and 15.6% had ever practiced sex work outside the district.

Due to the small number of women who reported community violence, for the remaining analyses, workplace and community violence were combined into one category ‘workplace / community violence’. Amongst FSWs who experienced recent violence, socio-demographic and sex work characteristics differed by the perpetrator of violence (Table 2). Women who reported recent workplace/community violence were more likely to solicit clients from public places (53.7%), whereas women who reported recent domestic violence only were more likely to solicit clients by phone (53.9%). A higher median number of clients per week was reported among women who experienced workplace/community violence (9; IQR: 5-12) or violence by both domestic *and* workplace/community perpetrators (9; IQR: 6-15), and these women were more likely to have migrated for sex work compared with women who had experienced domestic violence only or no violence (Table 2). Women who reported recent violence by both domestic *and* workplace/community perpetrators had the lowest mean age at start of sex work (25.4 years) and lowest mean age at first sex (15.4 years).

Table 2 Socio-demographic and sex work characteristics of FSWs in Shimoga and Bangalore and associations with violence by perpetrator

Characteristic	Overall	Recent violence by perpetrator

For peer review only

			No violence	Domestic violence only	Workplace and/or community violence only	Domestic and workplace/ community violence	P value (chi square test)
			%(n=727)	%(n=216)	%(n=80)	%(n=69)	
Age, years	<25	13.1	12.2	13.3	22.0	12.7	0.18
	25-29	22.4	20.9	24.6	21.9	36.1	
	30-39	45.2	44.6	47.4	41.0	42.7	
	40+	19.3	22.4	14.6	15.1	8.5	
	Mean	32.9	33.4	32.2	31.1	30.8	
Literacy	Illiterate	54.5	56.2	56.2	37.8	57.9	0.07
Marital Status	Lives alone	44.2	53.3	8.3	78.7	18.1	<0.0001
	Lives with partner other than husband	4.5	4.4	4.6	5.8	4.6	
	Married and lives with husband	51.2	42.3	87.0	15.5	76.2	
Regular partner	Yes	66.2	58.3	95.1	46.6	88.2	<0.0001
Number of children	0	9.7	8.7	7.3	22.5	12.2	0.03
	1-2	60.3	62.0	60.1	57.8	53.9	
	3+	30.1	29.4	32.6	19.7	34.0	
	Mean	2.0	2.0	2.2	1.7	2.0	
District	Bangalore	50.8	54.0	42.1	64.1	50.2	0.022
	Shimoga	49.3	46.0	57.9	35.9	49.8	
Additional income to sex work[1]	Yes	66.1	67.5	67.7	58.5	59.5	0.31
Age at first sex (years)	<15	48.2	49.4	44.9	38.9	64.6	0.07
	15+	51.8	50.6	55.1	61.1	35.4	
	Mean	16.1	16.1	16.3	16.6	15.4	
Age started sex work (years)	<20	5.5	5.6	4.8	8.2	5.7	0.056
	20-24	25.2	22.5	26.8	31.1	37.6	
	25-29	29.0	26.8	33.5	30.9	32.5	
	30+	40.3	45.1	35.0	29.8	24.3	
	Mean	28.3	28.9	27.7	26.9	25.4	
Place of solicitation of sex work	Home	7.4	8.4	7.9	3.1	4.7	0.0008
	Rented room/lodge/brothel	3.4	2.6	6.7	0.6	2.4	
	Public place/tamasha/other	32.5	28.3	31.5	53.7	43.4	
	Phone	56.7	60.7	53.9	42.6	49.4	
How much charged for sex with last client	400+	53.1	53.1	52.2	62.6	46.7	0.43
	Mean	459.3	469.8	442.5	458.5	422.3	

For peer review only - http://bmjopen.bmj.com/site/about/guidelines.xhtml

(rupees)							
Number of clients/ week	1-4	28.4	28.2	34.4	18.2	12.8	<0.0001
	5-9	45.0	46.6	47.3	37.7	42.0	
	10+	26.6	25.2	18.3	44.1	45.2	
	Median	6.0	6.0	6.0	9.0	9.0	
Migrant sex work (ever practiced sex work outside the district and/or in Mumbai)	Yes	15.6	12.6	11.9	39.9	25.8	<0.0001

Missing observations: [1] n=6(0.5%), [2] n=1(0.1%)

HIV/STI risk

Overall HIV prevalence was 8.2%, reactive syphilis 3.1% and high-titre syphilis 0.5%. In multivariate analysis, there was no evidence of an association between violence by perpetrator and either HIV (P-value: 0.27) or reactive syphilis (P-value: 0.76) (Table 3). However, there was strong evidence (P-value <0.0001) for an increased odds of high-titre syphilis infection amongst women who reported recent violence by both domestic *and* workplace/community perpetrators compared with women who reported no recent violence (aOR: 24.96; 95% CI: 5.94-96.70).

Self-report of STI symptoms (vaginal discharge/genital ulcers/abdominal pain not associated with menses) in the past year was higher amongst women who reported recent violence compared to FSWs who reported no violence. In multivariate analyses, there was strong evidence for an increased odds of STI symptoms in all categories of violence by perpetrator, with those who experienced violence by both domestic *and* workplace/community perpetrators having the highest odds of STI symptoms (aOR: 3.90; 95% CI: 2.10-7.26) (Table 3).

Condom Use

Recent violence by a specific perpetrator was associated with reduced condom use in that setting (Table 3). In adjusted analyses, any recent violence experience, regardless of the perpetrator, was associated with a significant reduction in reported condom use at last sex with occasional and regular clients. In multivariate analysis, recent violence experience by workplace perpetrators, or by domestic *and* workplace/community perpetrators, was significantly associated with reduced condom use with last occasional client and last regular client, compared with women reporting no recent violence. Overall, just one-fifth (19.5%) of FSWs reported condom use at last sex with a regular partner. Reported condom use with regular

For peer review only

For peer review only - http://bmjopen.bmj.com/site/about/guidelines.xhtml

partners was lower amongst women reporting recent domestic violence compared with women reporting no recent domestic violence, although this association did not remain significant in multivariate analyses.

Condom breakage at last sex was more likely among women who reported any recent violence (5.1%) compared to those who did not report recent violence (1.2%). In multivariate analysis there was strong evidence (P-value: 0.0001) for increased condom breakage among women who reported recent domestic violence (aOR: 3.72; 95%CI: 1.13-12.25), with the highest odds amongst women who reported violence by both domestic *and* workplace/community perpetrators (aOR: 19.29; 95%CI: 5.42-68.73).

Alcohol use

In univariate and adjusted analyses women who reported recent violence by workplace/community perpetrators (53.9%; aOR: 1.66; 95%CI: 0.96-2.84; P value: 0.024) and by both domestic *and* workplace/community perpetrators (56.0%; aOR: 2.16; 95%CI: 1.19-3.92, P-value: 0.024) were more likely to report either themselves, their client or both being under the influence of alcohol at last sex compared to women who reported no violence or domestic violence only.

Programme Exposure

Women who reported any recent violence were more likely to have visited an STI clinic in the last six months (44.5%) compared to those who did not report recent violence (27.8%) with the highest aOR amongst those who reported recent violence by both domestic *and* workplace/community perpetrators (aOR: 3.18; 95% CI: 1.68-6.03). Women who had experienced any recent violence (96.9%) were more likely to have had contact with a peer educator in the past month compared to women who had not experienced recent violence (92.0%), with some evidence for this association in multivariate analyses (aOR: 2.22; 95% CI: 0.98-5.00; P-value: 0.055).

For peer review only - http://bmjopen.bmj.com/site/about/guidelines.xhtml

Table 3 Violence by perpetrator and associations with HIV/STI prevalence and sexual risk behaviours

		Recent violence from any perpetrator			Violence by perpetrator (reference group: no recent violence)			
		**No recent violence** *%(n=727)*	**Any recent violence** *%(n=365)*	**P value**	**Domestic violence only** *%(n=216)*	**Workplace and/or community violence only** *%(n=80)*	**Domestic *and* workplace or community violence** *%(n=69)*	**P value***
HIV	%	8.1	6.1		2.5	13.4	8.9	
	Crude OR	1.0	0.73(0.42-1.27)	0.26	0.28(0.11-0.73)	1.75(0.88-3.5.0)	1.11(0.40-3.38)	0.022
	Adjusted OR	1.0	0.82(0.44-1.53)	0.53	0.40(0.15-1.09)	1.16(0.55-2.44)	1.32(0.41-4.29)	0.27
Reactive syphilis[1]	%	3.4	2.9		1.5	6.2	3.5	
	Crude OR	1.0	0.87(0.40-1.91)	0.74	0.42(0.09-2.02)	2.04(0.60-6.89)	1.12(0.30-4.22)	0.60
	Adjusted OR	1.0	1.27(0.68-2.38)	0.46	1.14(0.30-4.46)	1.17(0.57-2.40)	2.04(0.53-7.81)	0.76
High titre syphilis (recent syphilis)	%	0.38	0.64		0	0.9	2.5	
	Crude OR	1.0	1.70(0.36-8.03)	0.50	-	2.36(0.25-22.07)	6.74(1.15-39.58)	0.11
	Adjusted OR	1.0	2.22(0.54-9.17)	0.27	-	2.27(0.26-19.8)	24.96(5.94-96.70)	<0.0001
STI symptoms in past 12 months (vaginal discharge, lower abdominal pain not associated with menses and/or genital ulcer)	%	30.7	48.9		41.5	57.3	63.3	
	Crude OR	1.0	2.16(1.61-2.89)	<0.0001	1.60(1.11-2.31)	3.03(1.77-5.18)	3.90(2.18-6.95)	<0.0001
	Adjusted OR	1.0	2.27(1.66-3.09)	<0.0001	1.87(1.24-2.81)	2.41(1.40-4.17)	3.90(2.10-7.26)	<0.0001
Condom use last sex with occasional client[2]	%	97.5	94.5		97.2	91.6	91.0	
	Crude OR	1.0	0.45(0.21-0.96)	0.038	0.87(0.29-2.63)	0.28(0.10-0.74)	0.26(0.09-0.75)	0.0073
	Adjusted OR	1.0	0.39(0.19-0.83)	0.014	1.03(0.33-3.28)	0.20(0.07-0.52)	0.22(0.06-0.81)	0.0001

For peer review only - http://bmjopen.bmj.com/site/about/guidelines.xhtml

Condom use last sex with regular client[3]	%	93.0	88.2		92.6	85.9	76.0	
	Crude OR	1.0	0.56(0.32-0.98)	0.043	0.94(0.44-2.01)	0.46(0.20-1.05)	0.24(0.11-0.50)	0.0012
	Adjusted OR	1.0	0.61(0.32-1.15)	0.12	1.25(0.54-2.90)	0.33(0.15-0.73)	0.25(0.10-0.59)	0.0003
Condom use at last sex with regular partner	%	23.1	13.8		12.2	27.8	10.4	
	Crude OR	1.0	0.53(0.32-0.89)	0.016	0.46(0.26-0.84)	1.29(0.51-3.22)	0.39(0.16-0.93)	0.012
	Adjusted OR	1.0	0.63(0.35-1.14)	0.13	0.79(0.42-1.51)	0.40(0.14-1.12)	0.48(0.14-1.67)	0.30
Condom breakage at last sex[4]	%	1.2	5.1		3.0	3.1	15.1	
	Crude OR	1.0	4.38(1.91-10.02)	0.0005	2.46(0.84-7.25)	2.60(0.62-10.9)	14.3(5.10-40.30)	<0.0001
	Adjusted OR	1.0	4.32(1.74-10.73)	0.0017	3.72(1.13-12.25)	1.71(0.36-8.20)	19.29(5.42-68.73)	0.0001
Either client, FSW or both under the influence of alcohol at last sex	%	35.8	42.4		34.1	53.9	56.0	
	Crude OR	1.0	1.32(0.99-1.76)	0.058	0.93(0.661.30)	2.09(1.24-3.52)	2.28(1.31-3.99)	0.0015
	Adjusted OR	1.0	1.29(0.09-1.77)	0.12	0.97(0.66-1.42)	1.66(0.96-2.84)	2.16(1.19-3.92)	0.024
Visited an STI clinic in past six months for STI symptoms[5]	%	27.8	44.5		39.0	51.6	53.2	
	Crude OR	1.0	2.08(1.49-2.92)	<0.0001	1.66(1.10-2.52)	2.77(1.61-4.78)	2.95(1.61-5.43)	<0.0001
	Adjusted OR	1.0	2.28(1.59-3.27)	<0.0001	2.04(1.28-3.24)	2.32(1.35-3.97)	3.18(1.68-6.03)	0.0001
Had contact with a peer educator in the last month[6]	%	92.0	96.9		96.3	100	95.2	
	Crude OR	1.0	2.74(1.24-6.07)	0.013	2.27(0.86-6.00)	-	1.73(0.55-5.43)	0.20
	Adjusted OR	1.0	2.22(0.98-5.00)	0.055	1.75(0.63-4.90)	-	1.18(0.36-3.92)	0.56

Models adjusted for age, district, marital status, migrant sex work, place of selling sex and having an income other than sex work;
Missing observations: [1] n=1 (0.1%), [2] n=1(0.1%), [3] n=1(0.1%), [4] n=3(0.3%), [5] n=159 (14.3%), [6] n=22(2.0%); *Adjusted Wald test: tests the null hypothesis that the co-efficients (categories of exposure to violence) are equal to zero

For peer review only - http://bmjopen.bmj.com/site/about/guidelines.xhtml

DISCUSSION

This is the first study globally, to our knowledge, to examine violence experience among FSWs by perpetrators in the workplace, community and home, and associations with biological outcomes and HIV/STI risk behaviours. We found a high prevalence of violence from a range of perpetrators experienced by FSWs in this setting in India, with recent domestic violence more commonly reported than violence by workplace or community perpetrators. Additionally, we found that HIV/STI risk differed by perpetrator of violence and was highest amongst women who reported recent violence from multiple perpetrators; women reporting violence by domestic *and* workplace/community perpetrators compared to women reporting violence from domestic perpetrators only and workplace/community perpetrators only, were significantly more likely to have high-titre syphilis infection and had the highest odds of recent STI symptoms, condom breakage at last sex, alcohol use at last sex and no condom use at last sex with regular clients. This study is the first of its kind to show that increased STI prevalence and HIV/STI risk among FSWs is associated with experience of violence from multiple perpetrators. It also adds to a growing body of research globally, reporting the burden and range of perpetrators of violence among FSWs.

The pathways between violence exposure and increased HIV/STI risk are complex. Theories of risk pathways include HIV/STI risk associated with forced sex with an HIV-infected partner, women fearing violence if they request condom use, and increased high-risk behaviours as a result of the psychological impact of sexual/physical abuse[2 29]. In South Africa, among women from the general population increasing frequency of violence, for example reporting many versus one episode of violence, has been associated with increased odds of HIV infection[30 31], suggesting a 'dose-response' effect between violence and HIV/STI risk. Although our study did not measure frequency of violence, it is possible that women who experience violence from multiple perpetrators experience more violence overall than those who experience violence from domestic or workplace perpetrators only. This may help to explain the increased STI prevalence and sexual risk behaviours amongst women in our study who reported violence from multiple perpetrators.

Despite the high rates of domestic violence, our study findings suggest that violence by workplace/community perpetrators is more important for increasing sexual risk behaviours overall and during sex work, compared with domestic violence. Although a previous study with FSWs in India reported an association between husband-

For peer review only - http://bmjopen.bmj.com/site/about/guidelines.xhtml

perpetrated violence and reduced condom use with clients[25], in our study, we found no associations between domestic violence and sexual risk behaviours in the workplace, such as condom use with clients. However, women in our study who reported domestic violence only did have increased odds of STI symptoms and condom breakage at last sex compared to women who did not report any recent violence, suggesting that domestic violence is associated with some level of increased HIV/STI risk.

A recent systematic review of domestic violence among women in India estimated the median prevalence of lifetime and domestic violence in the past year was 41% and 30%, respectively[32]. In our study FSWs reported a much higher prevalence of lifetime (60.1%) violence and similar rates of recent domestic violence (27.1%). These high levels of domestic violence need to be addressed to reduce impacts on physical and psychological health[33]. So far, HIV prevention programmes with FSWs have focused mainly on reducing workplace violence[7 8 21] and improving condom use with clients[26]. Although there are examples of successful interventions to reduce domestic violence in women in the general population[34], the efficacy of such interventions among FSWs is unknown. A cluster RCT with FSWs, in Karnataka India, aimed at reducing IPV and improving condom use with their lover/husband is currently being assessed, and is the first of its kind to address domestic violence among FSWs[35].

Prevalence of recent workplace violence was relatively high (11.1%) despite the success of recent violence interventions in Karnataka[7], with clients the major perpetrators. Reported community violence was low (4.1%) compared to violence from other perpetrators. However, FSWs may be at greater risk of violence from community perpetrators compared to women in the general population, due to stigma and dangerous working environments. An important finding was the strong association between having experienced violence by both domestic *and* workplace/community perpetrators and increased odds of high-titre syphilis, demonstrating biological evidence of increased STI risk. As high-titre syphilis infection indicates recent infection, the direction of the association is more plausible compared to measures of chronic STI infection (HIV and reactive syphilis). Unfortunately in IBBA R3, FSWs were not tested for other incident STIs, due to budget constraints, although violence has been associated with gonorrhea in previous IBBAs[7]. Self-reported STI symptoms were strongly associated with violence from all perpetrators with the highest odds amongst those who reported both

For peer review only - http://bmjopen.bmj.com/site/about/guidelines.xhtml

domestic *and* workplace/community violence. Although this may indicate STI infection in some cases, self-reported STI symptoms are not a reliable indicator of biological infection[36]. Vaginal discharge in women in India has been linked to depression and psychosocial stress, which may partly explain this association[37]. The reduced odds of condom use with clients amongst women who reported violence by workplace/community perpetrators and by both domestic *and* workplace/community perpetrators, but not domestic violence, indicates that the association between violence and HIV/STI risk may be driven by the environment in which the violence occurs (although this is based on the assumption that different perpetrators are associated with particular environments).

The finding that FSWs who report recent violence have higher STI clinic attendance and recent contact with a peer educator reflects positively on the HIV/STI prevention programme in Karnataka, suggesting recent experience of violence does not hinder women from accessing services. In this study, having experienced workplace/community violence and both domestic *and* workplace/community violence was associated with alcohol use at last sex. Having experienced violence can lead to increased alcohol consumption as a coping mechanism[19 21]. Alternatively being under the influence of alcohol may increase vulnerability to violence and arrest[19].

This study had strengths and limitations. Although previous research has examined IPV and workplace violence among FSWs[25], none have included community violence or examined associations with biological STI infection. Only one previous study in Soweto, South Africa has reported on the prevalence of violence experience from multiple perpetrators among FSWs[10], but this study did not examine associations with HIV/STI prevalence or risk behaviours. To our knowledge, our study is the first to demonstrate increased prevalence of STI infection and sexual risk behaviours among FSWs who experience violence from multiple perpetrators, compared with FSWs who report either no recent violence, or recent violence from domestic or workplace perpetrators only. Although the data were collected in 2011, they remain the most recent data on HIV/STI prevalence and risk behaviours among FSWs in Karnataka. In addition, this is one of the few datasets available globally, which assesses exposure to both workplace and domestic violence among FSWs, as well as biological and behavioural markers of HIV and STI risk and infection. Other important strengths were the robust sampling strategy and the reasonably large sample size. With cross-sectional data, it is not possible to ascertain the direction of

For peer review only - http://bmjopen.bmj.com/site/about/guidelines.xhtml

association for some outcomes or infer causality. Reporting bias may have contributed to over-reporting of certain outcomes (such as condom use) while more stigmatized and sensitive topics (such as alcohol consumption and violence) may have been under-reported. The categorisation of violence by perpetrators was based on crude definitions, which likely do not reflect the fluidity of relationships and environments in which the violence occurs. For example, women who sell sex at home may experience domestic and workplace violence in one physical environment while the definition between regular client and lover/partner can become blurred, with clients becoming lovers and vice versa. Some associations may have been due to chance, particularly for outcomes with small numbers and wide confidence intervals, such as high titre syphilis infection. Additionally overlaps in confidence intervals between the exposure categories, indicate there is uncertainty in whether there is a true difference in risk by perpetrator of violence. If the WHO standardized 13-item violence questionnaire, which has been shown to yield higher response rates[8], had also been used for non-partners, it might have increased reporting of violence from workplace and community perpetrators. There was a discrepancy in the timeframe of between recent non-partner physical violence (past 6 months) and recent non-partner sexual violence/intimate partner violence (past 12 months), which could have led to under-reporting of non-partner physical violence.

Despite these limitations, the findings of this study have important implications for HIV/STI prevention among FSWs. Violence against FSWs across both domestic, workplace and community settings needs to be addressed through integrated, comprehensive HIV programmes to enforce their human right to be able to live and work without fear for their safety.

For peer review only

Contributors:

AB conducted the analyses, and wrote the first draft of the manuscript. RP supervised the analyses and reviewed the article. RP, SI, HLM, LP, JB, and SM contributed to the study design and reviewed the article. TSB conceptualised the study, supervised the analyses, and reviewed the article. The authors thank the women who participated in this study.

Funding:

This study was supported by the India AIDS Initiative (*Avahan*) of the Bill & Melinda Gates Foundation, grant no. OPP52138. Tara Beattie, Ravi Prakash, Lucy Platt and Shajy Isac were supported by the UK Department for International Development (DFID) as part of STRIVE, a 6-year programme of research and action devoted to tackling the structural drivers of HIV (http://STRIVE.lshtm.ac.uk/). Tara Beattie is supported by a British Academy Fellowship. The views expressed herein are those of the authors and do not necessarily reflect the official policy or position of the Bill and Melinda Gates Foundation, UK DFID or the British Academy.

Competing Interests:

None

Data Sharing Statement:

No additional data are available.

For peer review only

REFERENCES

1. Gupta GR, Parkhurst JO, Ogden JA, et al. Structural approaches to HIV prevention. *Lancet* 2008;372(9640):764-75. doi: 10.1016/s0140-6736(08)60887-9 [published Online First: 2008/08/09]
2. Dunkle KL, Decker MR. Gender-based violence and HIV: reviewing the evidence for links and causal pathways in the general population and high-risk groups. *Am J Reprod Immunol* 2013;69 Suppl 1:20-6. doi: 10.1111/aji.12039
3. Deering KN, Amin A, Shoveller J, et al. A systematic review of the correlates of violence against sex workers. *Am J Public Health* 2014;104(5):e42-54. doi: 10.2105/ajph.2014.301909 [published Online First: 2014/03/15]
4. Devries KM, Mak JYT, García-Moreno C, et al. The Global Prevalence of Intimate Partner Violence Against Women. *Science* 2013;340(6140):1527-28. doi: 10.1126/science.1240937
5. Baral S, Beyrer C, Muessig K, et al. Burden of HIV among female sex workers in low-income and middle-income countries: a systematic review and meta-analysis. *The Lancet Infectious Diseases* 2012;12(7):538-49.
6. Decker MR, Crago A-L, Chu SKH, et al. Human rights violations against sex workers: burden and effect on HIV. *The Lancet*;385(9963):186-99. doi: 10.1016/S0140-6736(14)60800-X
7. Beattie TS, Bhattacharjee P, Isac S, et al. Declines in violence and police arrest among female sex workers in Karnataka state, south India, following a comprehensive HIV prevention programme. *J Int AIDS Soc* 2015;18:20079. doi: 10.7448/IAS.18.1.20079
8. Beattie TS, Bhattacharjee P, Ramesh BM, et al. Violence against female sex workers in Karnataka state, south India: impact on health, and reductions in violence following an intervention program. *BMC Public Health* 2010;10:476. doi: 10.1186/1471-2458-10-476
9. Schwitters A, Swaminathan M, Serwadda D, et al. Prevalence of rape and client-initiated gender-based violence among female sex workers: Kampala, Uganda, 2012. *AIDS and behavior* 2015;19 Suppl 1:S68-76. doi: 10.1007/s10461-014-0957-y [published Online First: 2014/11/30]
10. Coetzee J, Gray GE, Jewkes R. Prevalence and patterns of victimization and polyvictimization among female sex workers in Soweto, a South African township: a cross-sectional, respondent-driven sampling study. *Glob Health Action* 2017;10(1):1403815. doi: 10.1080/16549716.2017.1403815 [published Online First: 2017/12/07]
11. Pando MA, Coloccini RS, Reynaga E, et al. Violence as a barrier for HIV prevention among female sex workers in Argentina. *PLoS ONE [Electronic Resource]* 2013;8(1):e54147.
12. Decker MR, Pearson E, Illangasekare SL, et al. Violence against women in sex work and HIV risk implications differ qualitatively by perpetrator. *BMC Public Health* 2013;13:876.
13. Decker MR, McCauley HL, Phuengsamran D, et al. Violence victimisation, sexual risk and sexually transmitted infection symptoms among female sex workers in Thailand. *Sexually Transmitted Infections* 2010;86(3):236-40.
14. Tounkara FK, Diabate S, Guedou FA, et al. Violence, condom breakage, and HIV infection among female sex workers in Benin, West Africa. *Sexually Transmitted Diseases* 2014;41(5):312-8.
15. Bradley J, Rajaram S, Moses S, et al. Female sex worker client behaviors lead to condom breakage: a prospective telephone-based survey in Bangalore, South India. *AIDS & Behavior* 2013;17(2):559-67.

16. Choi SY, Chen KL, Jiang ZQ. Client-perpetuated violence and condom failure among female sex workers in southwestern China. *Sexually Transmitted Diseases* 2008;35(2):141-6.
17. Shannon K, Strathdee SA, Shoveller J, et al. Structural and environmental barriers to condom use negotiation with clients among female sex workers: implications for HIV-prevention strategies and policy. *Am J Public Health* 2009;99(4):659-65. doi: 10.2105/ajph.2007.129858 [published Online First: 2009/02/07]
18. Li Q, Li X, Stanton B. Alcohol Use Among Female Sex Workers and Male Clients: An Integrative Review of Global Literature. *Alcohol and Alcoholism (Oxford, Oxfordshire)* 2010;45(2):188-99. doi: 10.1093/alcalc/agp095
19. Mbonye M, Rutakumwa R, Weiss H, et al. Alcohol consumption and high risk sexual behaviour among female sex workers in Uganda. *African journal of AIDS research : AJAR* 2014;13(2):145-51. doi: 10.2989/16085906.2014.927779 [published Online First: 2014/09/02]
20. Chersich MF, Bosire W, King'ola N, et al. Effects of hazardous and harmful alcohol use on HIV incidence and sexual behaviour: a cohort study of Kenyan female sex workers. *Global Health* 2014;10:22.
21. Biradavolu MR, Burris S, George A, et al. Can sex workers regulate police? Learning from an HIV prevention project for sex workers in southern India. *Social Science & Medicine* 2009;68(8):1541-47. doi: http://dx.doi.org/10.1016/j.socscimed.2009.01.040
22. Gurnani V, Beattie TS, Bhattacharjee P, et al. An integrated structural intervention to reduce vulnerability to HIV and sexually transmitted infections among female sex workers in Karnataka state, south India. *BMC Public Health* 2011;11:755. doi: 10.1186/1471-2458-11-755
23. Panchanadeswaran S, Johnson SC, Sivaram S, et al. Intimate partner violence is as important as client violence in increasing street-based female sex workers' vulnerability to HIV in India. *International Journal of Drug Policy*;19(2):106-12.
24. Deering KN, Bhattacharjee P, Bradley J, et al. Condom use within non-commercial partnerships of female sex workers in southern India. *BMC Public Health*;11 Suppl 6:S11.
25. Reed E, Erausquin JT, Groves AK, et al. Client-perpetrated and husband-perpetrated violence among female sex workers in Andhra Pradesh, India: HIV/STI risk across personal and work contexts. *Sexually Transmitted Infections* 2016 doi: 10.1136/sextrans-2015-052162
26. Ramesh BM, Beattie TS, Shajy I, et al. Changes in risk behaviours and prevalence of sexually transmitted infections following HIV preventive interventions among female sex workers in five districts in Karnataka state, south India. *Sexually Transmitted Infections* 2010;86 Suppl 1:i17-24.
27. Reza-Paul S, Beattie T, Syed HU, et al. Declines in risk behaviour and sexually transmitted infection prevalence following a community-led HIV preventive intervention among female sex workers in Mysore, India. *AIDS (London, England)* 2008;22 Suppl 5:S91-100. doi: 10.1097/01.aids.0000343767.08197.18 [published Online First: 2009/01/07]
28. World Health Organisation. WHO multi-country study on women's health and domestic violence against women: Initial results on prevalence, health outcomes and women's responses. Geneva, 2005.
29. Ulibarri MD, Strathdee SA, Lozada R, et al. Prevalence and correlates of client-perpetrated abuse among female sex workers in two Mexico-U.S. border cities. *Violence Against Women* 2014;20(4):427-45.
30. Jewkes RK, Dunkle K, Nduna M, et al. Intimate partner violence, relationship power inequity, and incidence of HIV infection in young women in South Africa: a cohort study. *Lancet* 2010;376(9734):41-8.

For peer review only - http://bmjopen.bmj.com/site/about/guidelines.xhtml

31. Dunkle KL, Jewkes RK, Brown HC, et al. Gender-based violence, relationship power, and risk of HIV infection in women attending antenatal clinics in South Africa. *Lancet* 2004;363(9419):1415-21. doi: 10.1016/s0140-6736(04)16098-4 [published Online First: 2004/05/04]
32. Kalokhe A, del Rio C, Dunkle K, et al. Domestic violence against women in India: A systematic review of a decade of quantitative studies. *Global Public Health* 2016:1-16. doi: 10.1080/17441692.2015.1119293
33. World Health Organisation, London School of Hygiene & Tropical Medicine, South African Medical Research Council. Global and regional estimates of violence against women: prevalence and health effects of intimate partner violence and non-partner sexual violence, 2013.
34. Pronyk PM, Hargreaves JR, Kim JC, et al. Effect of a structural intervention for the prevention of intimate-partner violence and HIV in rural South Africa: a cluster randomised trial. *Lancet* 2006;368(9551):1973-83. doi: 10.1016/s0140-6736(06)69744-4 [published Online First: 2006/12/05]
35. Beattie TS, Isac S, Bhattacharjee P, et al. Reducing violence and increasing condom use in the intimate partnerships of female sex workers: study protocol for Samvedana Plus, a cluster randomised controlled trial in Karnataka state, south India. *BMC Public Health* 2016;16:660. doi: 10.1186/s12889-016-3356-7
36. Vishwanath S, Talwar V, Prasad R, et al. Syndromic management of vaginal discharge among women in a reproductive health clinic in India. *Sexually Transmitted Infections* 2000;76(4):303-06. doi: 10.1136/sti.76.4.303
37. Patel V, Pednekar S, Weiss H, et al. Why do women complain of vaginal discharge? A population survey of infectious and pyschosocial risk factors in a South Asian community. *Int J Epidemiol* 2005;34(4):853-62. doi: 10.1093/ije/dyi072 [published Online First: 2005/04/19]

FIGURES

Figure 1 Proportional venn diagram showing overlapping of physical and/or sexual violence experiences among FSWs by perpetrator

APPENDICES

See supplementary files for Appendix A (violence questionnaire)

For peer review only

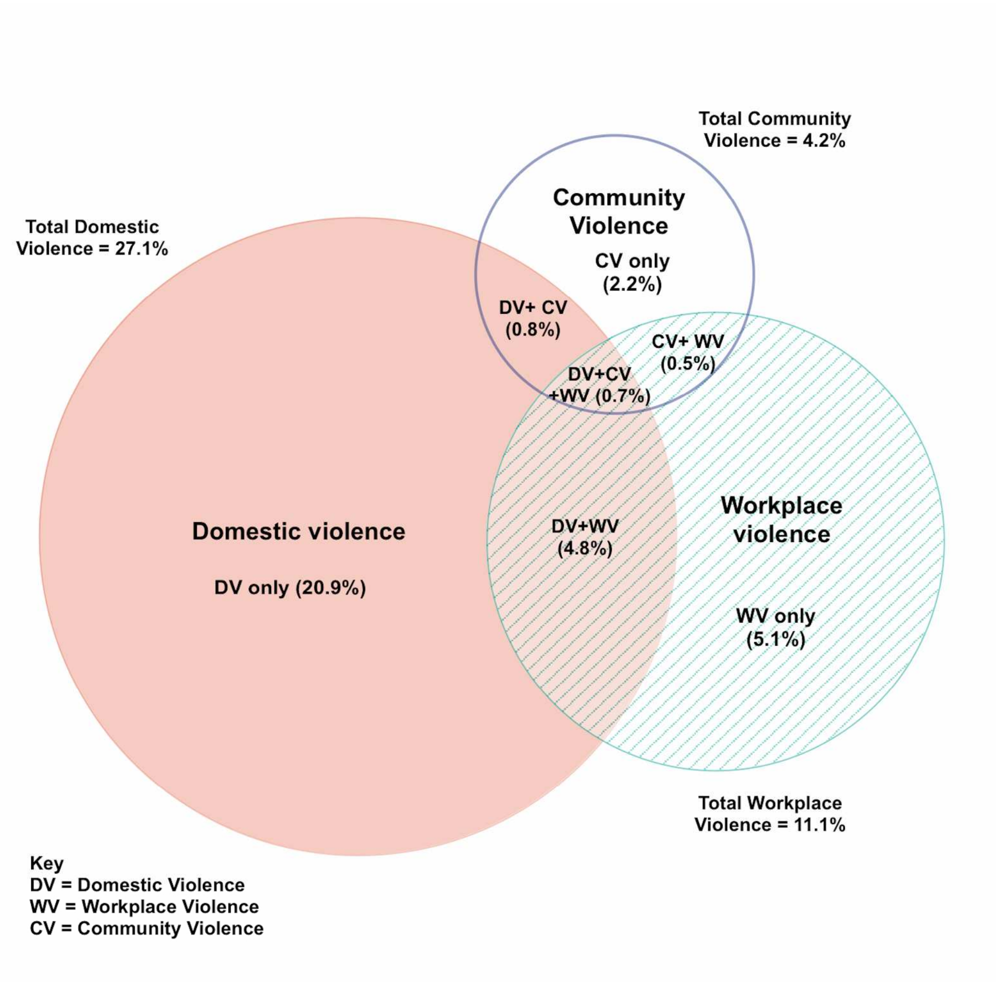

Figure 1 Proportional venn diagram showing overlapping of physical and/or sexual violence experiences among FSWs by perpetrator

260x255mm (300 x 300 DPI)

Appendix A: IBBA violence questionnaire

Physical and sexual violence from a husband/main partner based on WHO operational definitions of violence	Q717b: A. Has any husband or main partner that you have lived with ever done following things to you?<br><br>a) pushed you, shaken you, or thrown something at you?<br><br>b) slapped or shoved you?<br><br>c) hit you with his fist or something else that could hurt you?<br><br>d) kicked you, dragged you or beat you up?<br><br>e) tried to choke you or burn you on purpose<br><br>f) threatened to use or actually used a knife, gun or any other weapon?<br><br>g) physically forced to have sex with him even when you did not want.<br><br>h) used threats of violence or rejection to forced you to have sex with him when you did not want to?<br><br>B. How often has this happened during the last 12 months: often, only sometimes, or not at all?

For peer review only

STROBE checklist for manuscript: Violence experience by perpetrator and associations with HIV/STI risk and infection: a cross-sectional study among female sex workers in Karnataka, south India

STROBE Statement—Checklist of items that should be included in reports of ***cross-sectional studies***

	Item No	Recommendation	Reported on page #
**Title and abstract**	1	(*a*) Indicate the study's design with a commonly used term in the title or the abstract	2
		(*b*) Provide in the abstract an informative and balanced summary of what was done and what was found	2, 3
**Introduction**			
Background/rationale	2	Explain the scientific background and rationale for the investigation being reported	4, 5
Objectives	3	State specific objectives, including any prespecified hypotheses	4,5
**Methods**			
Study design	4	Present key elements of study design early in the paper	5, 6
Setting	5	Describe the setting, locations, and relevant dates, including periods of recruitment, exposure, follow-up, and data collection	6
Participants	6	(*a*) Give the eligibility criteria, and the sources and methods of selection of participants	6
Variables	7	Clearly define all outcomes, exposures, predictors, potential confounders, and effect modifiers. Give diagnostic criteria, if applicable	7
Data sources/ measurement	8*	For each variable of interest, give sources of data and details of methods of assessment (measurement). Describe comparability of assessment methods if there is more than one group	7
Bias	9	Describe any efforts to address potential sources of bias	7
Study size	10	Explain how the study size was arrived at	6
Quantitative variables	11	Explain how quantitative variables were handled in the analyses. If applicable, describe which groupings were chosen and why	7
Statistical methods	12	(*a*) Describe all statistical methods, including those used to control for confounding	7
		(*b*) Describe any methods used to examine subgroups and interactions	7
		(*c*) Explain how missing data were addressed	
		(*d*) If applicable, describe analytical methods taking account of sampling strategy	7
		(*e*) Describe any sensitivity analyses	
**Results**			
Participants	13*	(a) Report numbers of individuals at each stage of study—eg numbers potentially eligible, examined for eligibility, confirmed eligible, included in the study, completing follow-up, and analysed	8
		(b) Give reasons for non-participation at each stage	
		(c) Consider use of a flow diagram	
Descriptive data	14*	(a) Give characteristics of study participants (eg demographic, clinical, social) and information on exposures and potential confounders	9

For peer review only - http://bmjopen.bmj.com/site/about/guidelines.xhtml

		(b) Indicate number of participants with missing data for each variable of interest	9, 10, 14
Outcome data	15*	Report numbers of outcome events or summary measures	11, 12, 13, 14
Main results	16	(*a*) Give unadjusted estimates and, if applicable, confounder-adjusted estimates and their precision (eg, 95% confidence interval). Make clear which confounders were adjusted for and why they were included	11, 12, 13, 14
		(*b*) Report category boundaries when continuous variables were categorized	9, 10
		(*c*) If relevant, consider translating estimates of relative risk into absolute risk for a meaningful time period	
Other analyses	17	Report other analyses done—eg analyses of subgroups and interactions, and sensitivity analyses	
**Discussion**			
Key results	18	Summarise key results with reference to study objectives	15, 17
Limitations	19	Discuss limitations of the study, taking into account sources of potential bias or imprecision. Discuss both direction and magnitude of any potential bias	16. 17
Interpretation	20	Give a cautious overall interpretation of results considering objectives, limitations, multiplicity of analyses, results from similar studies, and other relevant evidence	15-17
Generalisability	21	Discuss the generalisability (external validity) of the study results	15, 17
**Other information**			
Funding	22	Give the source of funding and the role of the funders for the present study and, if applicable, for the original study on which the present article is based	18

*Give information separately for exposed and unexposed groups.

Note: An Explanation and Elaboration article discusses each checklist item and gives methodological background and published examples of transparent reporting. The STROBE checklist is best used in conjunction with this article (freely available on the Web sites of PLoS Medicine at http://www.plosmedicine.org/, Annals of Internal Medicine at http://www.annals.org/, and Epidemiology at http://www.epidem.com/). Information on the STROBE Initiative is available at www.strobe-statement.org.

For peer review only

For peer review only - http://bmjopen.bmj.com/site/about/guidelines.xhtml
